# Optimization of automated sea-ice melt-pond-depth determination in ICESat-2 altimeter data with the Density-Dimension Algorithm for bifurcating sea-ice reflectors using airborne campaign data

**DOI:** 10.1017/jog.2026.10167

**Published:** 2026-05-19

**Authors:** Thomas Trantow, Ute Herzfeld, Mia Vanderwilt, Kutalmis Saylam, Nathan Kurtz, Huilin Han, Rachel Tilling

**Affiliations:** 1 Geomathematics, Remote Sensing and Cryospheric Sciences Laboratory, Department of Electrical, Computer and Energy Engineering, University of Colorado, Boulder, CO, USA; 2 Department of Computer Science, University of Colorado, Boulder, CO, USA; 3 Earth System Science Interdisciplinary Center, University of Maryland, College Park, MD, USA; 4 NASA Goddard Space Flight Center, Greenbelt, MD, USA; 5 Bureau of Economic Geology, Near Surface Observatory, Jackson School of Geosciences, University of Texas, Austin, TX, USA

**Keywords:** Arctic glaciology, laser altimetry, melt-surface, sea ice

## Abstract

Melt ponding on Arctic sea ice is a key indicator of the transition from a predominantly perennial to a seasonal sea-ice cover, yet quantitative data on pond depth remain limited. Here, we present the first analysis of melt-pond depth using Ice, Cloud, and land Elevation Satellite-2 (ICESat-2)’s Advanced Topographic Lidar Altimeter System (ATLAS). The Density-Dimension Algorithm for bifurcating sea-ice reflectors (DDA-bifurcate-seaice) automatically detects multiple surface returns in ICESat-2 photon data and estimates corresponding surface heights, enabling melt-pond-depth retrievals under varied noise conditions.

Airborne lidar and imagery collected during the NASA ICESat-2 Project Arctic Summer Sea Ice Campaign (July 2022) provide near-coincident observations used to evaluate and optimize the algorithm’s melt-pond detection. Evaluation of the melt-pond-depth quantile using Chiroptera data shows that the uniform value used in the ATL07 release 7 data product is near-optimal. We demonstrate DDA-bifurcate-seaice’s capability to detect a wide range of melt feature morphologies, including smooth or rough bottoms, ridge-adjacent ponds, partial drainage and seawater intrusion. To further improve depth determination, we propose a depth-quantile function that reduces bias and mean-squared error by a factor of 2.75 and 2.2, respectively. This work improves melt-pond-depth estimation using the DDA-seaice-bifurcate, supporting Arctic- and Antarctic-wide mapping in the ICESat-2/ATLAS experimental sea-ice melt-pond data product on ATL07 (release 7).

## 1. Introduction

As Arctic sea ice has repeatedly reached historic lows (Stroeve and others, 2012; Parkinson and DiGirolamo, 2021; Gilbert and Holmes, 2024), and the transition from a perennial to a seasonal ice cover appears imminent (Kwok, 2018; Arthun and others, 2021; Jahn and others, 2024), melt ponding has emerged as a key process in understanding this change. Ubiquitous on sea ice during the summer melting season, melt ponds play an important role in the energy balance of the Arctic climate system (Eicken and others, 2004; Perovich and Polashenski, 2012), and provide key parameters in modeling sea-ice evolution (e.g., Hunke and others (2013); Schröder and others (2014); Sterlin and others (2021)) where model predictions diverge in their 21st century projections (Stroeve and others, 2007; Notz and SIMIP Community, 2020; Sardana and others, 2025). Reliable observational datasets of melt-pond characteristics, particularly depth, are critical for quantifying and understanding the temporal and spatial evolution of melt-pond dynamics. However, comprehensive detection and characterization of melt ponds is challenging due to the complexity of the Arctic sea-ice environment, where melt ponds are closely interspersed with open water, deformed ice (e.g., ridges) and varying material properties across the ice, snow and meltwater.

The Advanced Topographic Laser Altimeter System (ATLAS), the micro-pulse photon counting lidar aboard NASA’s Ice, Cloud, and land Elevation Satellite-2 (ICESat-2), provides year-round sea-ice surface height and freeboard estimates (Kwok and others, 2014), allowing measurement and monitoring of the onset of melt in the Arctic and on melt-pond evolution (Tilling and others, 2020; Buckley and others, 2023; Herzfeld and others, 2023). However, prior to launch, there was uncertainty regarding the appearance of the ICESat-2 return signal when penetrating water and whether multiple surface layers could be detected. Consequently, the detection of melt ponds was not a design objective of the standard sea-ice algorithm—the ATLAS/ICESat-2 L3A Sea Ice Height product (ATL07; Kwok and others (2022, 2023)). As a result, melt ponds are not captured, contributing to increased uncertainty in ICESat-2 sea-ice measurements during the summer months (Tilling and others, 2020). ATL07 reports only a single surface height at limited resolution, tracking either the pond-top surface, the pond-bottom surface, or somewhere in between, depending on surface reflectance properties (Farrell and others, 2020). This variability indicates that ATL07 heights are unreliable in the presence of melt ponds and, consequently, cannot be used to estimate melt-pond characteristics such as pond depth and width.

To address the challenge of melt-pond detection and characterization in altimeter data, Herzfeld and others (2023) developed the Density-Dimension Algorithm for bifurcating sea-ice reflectors (DDA-bifurcate-seaice), which is driven by a set of algorithm-specific parameters. This auto-adaptive algorithm is applied to the *ATLAS/ICESat-2 L2A Global Geolocated Photon Data* (ATL03, Neumann and others (2022)) and can identify and track multiple surfaces with complex topography (e.g., pond tops and bottoms among ridges and open water) across the Arctic near the along-track shot spacing of ICESat-2 data, which is 0.7 m under clear-sky atmospheric conditions. Therefore, the DDA-bifurcate-seaice has the capacity to provide value-added datasets of sea-ice melt-pond depth that are currently unavailable.

The algorithm has been included in a regional study examining summer melt on multi-year sea ice (Buckley and others, 2023; Herzfeld and others, 2024). In Buckley and others (2023), the DDA-bifurcate-seaice melt-pond-depth retrievals are compared to the non-automated University of Maryland melt pond algorithm (UMD-MPA, Farrell and others (2020)). Across 113 ponds in the Lincoln Sea, the mean difference in depth estimates between the two algorithms is -0.04±0.22 m (DDA-bifurcate-seaice minus UMD-MPA).

Across large spatial domains, even modest differences in retrieved pond depths can propagate into substantial discrepancies in integrated meltwater volume. In Buckley and others (2023), the DDA-bifurcate-seaice identified 7329 melt ponds during the study period. When combined with a mean depth difference of 4 cm between the two retrieval methods and a mean pond area of 182 m${}^{2}$ derived from imagery averaged over June–August 2020, this depth bias translates to a total meltwater volume difference of 53 355 m${}^{3}$ between the two algorithms. This result highlights that small differences in mean depth estimates can yield large uncertainties in aggregate meltwater volume, underscoring the sensitivity of sea-ice meltwater estimates to the choice of depth retrieval method and associated parameters.

In this paper, the primary goal is to optimize DDA-bifurcate-seaice parameterization to improve the accuracy and precision of melt-pond-depth retrievals, and ultimately reduce uncertainty in Arctic-wide meltwater volume estimates. Specifically, we seek to optimize the melt-pond-depth quantile that governs melt-pond bottom height estimation and, consequently, melt-pond-depth retrieval. To accomplish this, we leverage the coincident high-resolution lidar and imagery from the NASA ICESat-2 Project Arctic Summer Sea Ice Campaign conducted 11–26 July 2022, particularly the University of Texas at Austin’s Chiroptera sensor (Saylam and others, 2023). Optimization of algorithm-specific parameters enhances melt-pond characterization from ICESat-2 data, and by incorporating the DDA-bifurcate-seaice algorithm into the ATL07 data product, this work contributes to the development of Arctic- and Antarctic-wide sea-ice melt-pond data products.

In the following sections, we first present an overview of the ICESat-2 ATLAS data and relevant data products (Section 2.1), followed by a description of the Chiroptera airborne campaign data (Section 2.2). We then outline the methods used to detect and characterize melt ponds in ICESat-2 photon height data using the DDA-bifurcate-seaice algorithm (Section 3.1), in the airborne lidar and imagery data (Section 3.2) and in the parameter optimization routine that integrates both datasets (Section 3.3). Results from the melt-pond-depth analysis, including ponds with diverse material and morphological characteristics, are presented in Section 4.1, along with the derived relationship between the depth parameterization and maximum melt-pond depth (Section 4.3). Finally, Section 5 provides a summary of the findings and discusses implications for Arctic-wide melt-pond-depth estimation.

## 2. Data

### 2.1. ICESat-2

Launched on 15 September 2018, the ICESat-2 mission provides continuous height measurements across the cryosphere using the micro-pulse photon-counting capabilities of the ATLAS instrument (Markus and others, 2017; Neumann and others, 2019). Operating at 532 nm (green light), ATLAS employs six beams, each delivering independent height estimates across-track. The beams are organized into three pairs of ‘strong’ and ‘weak’ beams (distinguished by transmit energy), spaced 3.3 km apart across-track. Within each pair, the strong and weak beams are separated by 90 m across-track and 2.5 km along-track. The detailed beam geometry of ATLAS is illustrated in figure 3 of Herzfeld and others (2021b).

ATLAS’s narrow transmit pulse length (<1.5 ns) produces footprints approximately 11 m in diameter on the ground (Neumann and others, 2019; Magruder and others, 2021). With a laser pulse repetition frequency of 10 kHz and a spacecraft velocity of ~7 km/s, individual footprints are spaced roughly 0.7 m apart along track. Accordingly, ATLAS delivers single-shot measurements every ~0.7 m along track, with substantial overlap between adjacent footprints (Neumann and others, 2019).

ICESat-2 orbits at a 92${}^{\circ }$ inclination, enabling height estimates up to 88${}^{\circ }$N/S, and follows a 91-day repeat cycle. A single measurement cycle is divided into 1387 unique orbits, each associated with a reference ground track (RGT). These imaginary lines, located between the middle beam pair (i.e., at nadir), ensure that ATLAS follows prescribed tracks to enable repeat measurements (Magruder and others, 2021).

#### 2.1.1. ATL03

The *ATLAS/ICESat-2 L2A Global Geolocated Photon Data* (ATL03) data product (Neumann and others, 2023) consists of raw photon data (photon height, latitude, longitude and time) for each beam, along with ancillary data, from which many higher-level ICESat-2 products are derived (Neumann and others, 2022). At the time of analysis, the ATL03 data were in their release 6 version (Rel006), with release 7 becoming publically available in summer 2025. Specific ATL03 data segments, or granules, are uniquely identified by their date and time of acquisition, RGT number and version/revision number, and are freely available as described in the Data Availability section at the end of this paper.

#### 2.1.2. ATL07

The *ATLAS/ICESat-2 L3A Sea Ice Height* (ATL07) data product (Kwok and others, 2023), also in release 6 during this analysis, provides along-track heights for sea ice and open water leads at varying length scales along with height statistics and apparent reflectance (Kwok and others, 2022). Data are provided along each of the six ATLAS beams, with along-track averages typically over segments of ~40 m. The ATL07 product is also publicly available via Earthdata or NSIDC.

### 2.2. NASA ICESat-2 Project Arctic Summer Sea Ice Campaign

**Figure 1.**
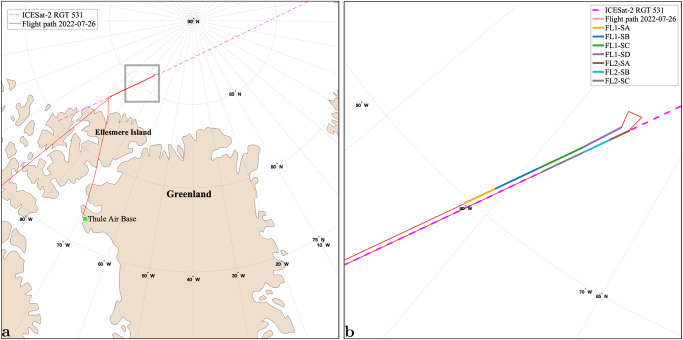
2022 NASA ICESat-2 Project Arctic Summer Sea Ice Campaign map for the 26 July 2022 flight. (a) 26 July 2022 flight path (solid red line) with the ICESat-2 RGT 531 (dashed magenta line, Granule: ATL03_20220726163210_05311604_006_02). Gray box indicates the location of subfigure b. (b) Data segments of the 26 July 2022 flight. Only data from Chiroptera data-swath 1 (FL1) are used in this analysis. 1 long description.

The NASA ICESat-2 Project Arctic Summer Sea Ice Campaign consisted of six science flights on NASA’s Johnson Space Center’s Gulfstream V (G-V) aircraft between 11 and 26 July 2022, operating out of Thule (Pituffik Space Base) in Northwest Greenland (Fig. 1). The primary goal of the campaign was to evaluate and enhance the retrieval of Arctic sea ice freeboard and melt-pond characteristics from ICESat-2 data during the summer melt season. During the campaign, two laser altimeter and visual imagery systems were operated: (1) NASA’s LVIS sensor (Blair and others, 1999) and (2) the University of Texas at Austin’s Chiroptera lidar/imager (Saylam and others, 2023). Coincident flights with ICESat-2 were carried out at high altitude using LVIS to provide broad coverage of the sea ice and ensure overlap with the ICESat-2 beams, and at low altitude using Chiroptera to capture fine-scale resolution measurements of sea-ice and melt-pond structure. In the present analysis, we utilize only the Chiroptera data.

#### 2.2.1. Chiroptera instrument system and data

The Chiroptera instrument system consists of a lidar and an optical imaging sensor. The Leica Chiroptera-4x airborne lidar system is comprised of a dual-frequency lidar scanner and a four-band high-resolution imager (Saylam and others, 2023, 2025). Chiroptera operates at low altitudes with respect to LVIS and during the campaign was flown at altitudes between 510 and 570 m, except during the last flight on 26 July 2022 where the altitude ranged between 586 and 1462 m.

Chiroptera’s two lidar scanners operate simultaneously at a green wavelength (515 nm) and a near-infrared (NIR) wavelength (1064 nm), emitting laser pulses with incidence angles ranging from 14${}^{\circ }$ to 20${}^{\circ }$. When sampling a melt pond, NIR pulses will reflect off the water surface, while green pulses penetrate the water column, where they slow and attenuate due to refraction and scattering. Green light will reflect from both the pond surface and the pond bottom, allowing estimates of pond depth at this single frequency. For our ICESat-2 comparison analysis here, we use only the 515 nm lidar data, hereafter referred to as Chiroptera-515 data.

Accuracy of the Chiroptera height measurements was assessed prior to the science flights using GNSS checkpoints on the Pituffik Space Base airport runway (Saylam and others, 2025). For Chiroptera-515 data, the absolute mean height difference was less than 1 cm with a coefficient of determination of $R^{2}$ = 0.99. Similarly, Chiroptera photon height measurements were compared to ICESat-2 ATL03 photon heights on the runway, where the absolute mean height difference was also smaller than 1 cm ($R^{2}$ = 0.99).

The analysis in this paper does not account for the refraction effects of photons traveling through water in both the Chiroptera and ICESat-2 datasets. This does not affect the depth comparisons, assuming the travel time difference between Chiroptera’s 515 nm light and ICESat-2’s 532 nm light is negligible. However, absolute melt-pond-depth estimates must account for the change in the speed of light in water, requiring a correction factor of approximately 0.75 (Buckley and others, 2020).

The Chiroptera system’s image sensor captures Red, Green, Blue (RGB) and NIR bands. For this analysis, we use a low-resolution version of the RGB imagery (0.5 m/pixel) to improve usability while maintaining a resolution comparable to ATLAS data (0.7 m). Individual image scenes from the 26 July 2022 flight cover approximately 500 × 500 m. All imagery is georeferenced to Universal Transverse Mercator coordinates, Zone 16N.

In the following comparison analysis, all coordinate data, including the latitude and longitude provided by the DDA-bifurcate-seaice output, are converted to WGS84/NSIDC Sea Ice Polar Stereographic North (EPSG:3413) with units in meters.

#### 2.2.2. Data limitations and subsets

Cloud cover and campaign logistics restricted the availability of useful datasets for evaluating DDA-bifurcate-seaice with airborne campaign data. Consequently, measurements useful for our study are only available from one of the six science flights, specifically the flight conducted on 26 July 2022 that collected data over multi-year sea ice in the Lincoln Sea (see Fig. 1). This is the only good-weather flight that collected both Chiroptera lidar and image data that were (near) coincident with an ICESat-2 track.

To manage the large volume of data collected during the flight, the Chiroptera dataset is divided into segments. Initially, the data are segmented based on their matching ICESat-2 beams: Chiroptera data-swath 1 (FL1) aligns with RGT 531 Beam 3, and data-swath 2 (FL2) aligns with RGT 531 Beam 2. Data-swath 3 (FL3) also aligns with Beam 2, although some necessary Chiroptera data products are unavailable for this subset. FL1 and FL2 are further divided into sections—A–D for FL1 and A–C for FL2—as shown in Fig. 1b.

This analysis utilizes data from FL1 only, which surveys RGT 531 Beam 3 between 8 m 26 s and 38 m 26 s after the associated ICESat-2 pass (Saylam and others, 2025). FL2 data are not included due to the high number of saturated returns in the near-nadir Beam 2, which complicates the determination of melt-pond characteristics in the photon data. Saturation of the ATLAS photon-counting receivers can arise from quasi-specular returns from flat water surfaces, resulting in the ‘dead-time effect’ (Tilling and others, 2020; Martino and others, 2023). Highly saturated returns, where many photon events arrive at the same time, will paralyze the detector, rendering it unable to record additional events during a dead time of 2.8–3.2 ns. In this case, the photon distribution exhibits a gap, with no photons immediately below the high-density returns from the flat surface. Once the detector dead time has elapsed, photons are recorded again below this gap, and their distribution can resemble the true flat surface, producing a false surface at a consistent depth determined by the detector dead time. Herzfeld and others (2023) discuss and demonstrate the effect of saturation on the detection capabilities of the DDA-bifurcate-seaice.

## 3. Methods

### 3.1. DDA-bifurcate-seaice

The DDA-bifurcate-seaice (Herzfeld and others, 2023) is part of the Density Dimension Algorithm (DDA) family that includes an algorithm for finding single ice-surface heights (DDA-ice, Herzfeld and others (2017, 2021b)), vegetation and canopy heights (DDA-sigma-veg, Herzfeld and others (2014)), and atmospheric layer boundaries for clouds and aerosols (DDA-atmos, Herzfeld and others (2021a)). The DDA-atmos is the operational algorithm for atmospheric layer characterization reported on the ATLAS/ICESat-2 atmospheric data product *Calibrated Backscatter Profiles and Atmospheric Layer Characteristics* (ATL09, Herzfeld and others (2022); Palm and others (2022)). The core of the DDA algorithmic approach is highly adaptable to other altimeter datasets, for example, the CALIOP-Density-Dimension Algorithm (CALIOP-DDA), which has been applied to the dual-frequency, multi-polarization CALIPSO atmospheric lidar data (Herzfeld and others, 2025).

The DDA-bifurcate-seaice extends the functionality of DDA-ice by incorporating the ability to bifurcate—splitting from tracking a single surface to tracking two distinct surfaces when distinct signals are detected—and then rejoining to track a single surface once the secondary signal is no longer present. The algorithm can handle situations where the stronger reflector is associated with either the lower or upper surface, accounting for differences in material and reflection properties.

**Figure 2.**
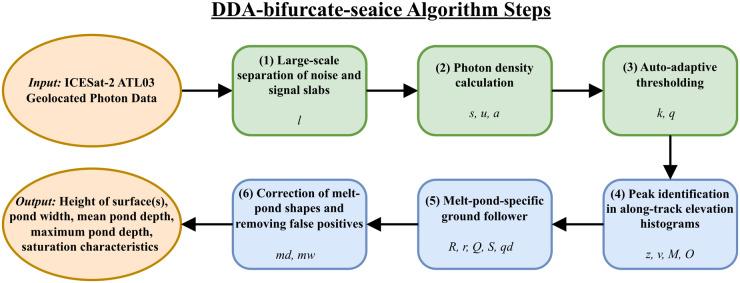
Flowchart of the DDA-bifurcate-seaice algorithmic steps. Steps are described in detail in Section 3.1.1 (Core DDA steps, green boxes) and Section 3.1.2 (Bifurcation-specific steps, blue boxes). Relevant algorithmic parameters, described in Table 1, for each step are given in italics at the bottom of each box. Figure 2 long description.

The algorithmic steps for DDA-bifurcate-seaice are discussed in detail in Sections 4E and 4F of Herzfeld and others (2023), as is the sensitivity study used to determine the algorithm-specific parameters for investigations of sea ice. Below, we summarize the core steps of the DDA algorithm family and outline the key components relevant to melt-pond-depth estimation within the bifurcation module of DDA-bifurcate-seaice, which is central to the focus of this study. The specific algorithmic parameters used in this analysis are given in Table 1, and a flowchart of the algorithmic steps is provided in Fig. 2.

**Table 1.** DDA parameters for the ICESat-2 Summer 2022 Arctic Sea Ice Campaign runs in this analysis. The parameters listed in the table from z onward apply specifically to the DDA-bifurcate-seaice algorithm. Units are provided in the second column for parameters with physical dimensions. Table 1 long description.

Symbol	Name/meaning	Value
s	Sigma, standard deviation of *rbf*-kernel (m)	3
u	Cutoff, number of standard deviations	2
a	Anisotropy (of kernel)	5*
q	Threshold quantile (of density)	0.15
k	Threshold bias offset (of density)	1
l	Slab-height (m)	30
R	Resolution of ground follower (m)	5*
r	Factor to refine the R parameter in case	2
	of rough surface as determined by S	
Q	Crevasse depth quantile	0.5
S	Standard deviation threshold	1.75
	of thresholded signal to trigger	
	small step size in ground follower (m)	
z	Horizontal histogram bin size (m)	25–70*
v	Vertical histogram bin size (m)	0.1
M	Minimum peak height in histograms	2*
O	Minimum peak prominence in histograms	2*
qd	Melt-pond-depth quantile	0.75**
md	Minimum depth of melt pond (m)	0.5
mw	Minimum amount of depth estimates	4
	required for melt-pond identification	

* Denotes parameters that differ from those in Table 1 of Herzfeld and others (2023), having been updated for more consistent melt-pond detection, as used in the derivation of the NSIDC DDA-bifurcate-seaice dataset (Herzfeld and others, 2024). *Default parameter value in the algorithm before the current analysis. A range of horizontal histogram bin sizes is used to optimally capture the individual widths of each sea-ice melt pond in the current investigation.

#### 3.1.1. Core DDA steps: density calculation and auto-adaptive thresholding

**Figure 3.**
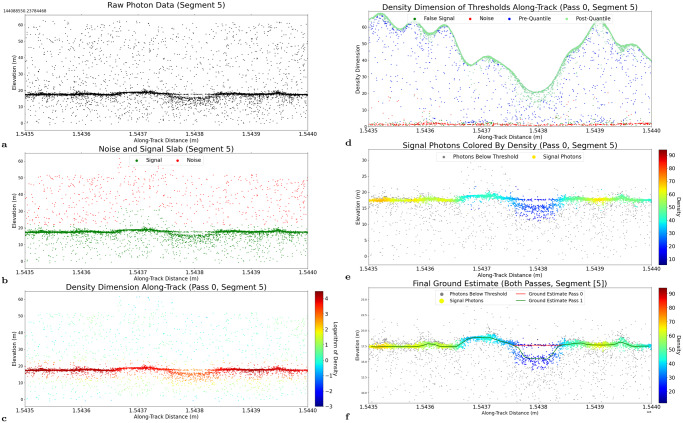
Steps of the DDA-bifurcate-seaice. (a) Raw ATL03 photon data. (b) Large-scale photon separation into signal (green) and noise (red) slabs. (c) Density of each photon in both signal and noise slabs. (d) Photon classification based on auto-adaptive thresholding procedure (note vertical axis here is density rather than elevation). (e) Thresholded signal photons as a result of Steps (1)–(3) in the algorithm. (f) Interpolated surface heights for top surface (red line) and bottom surface (green line) given by the melt-pond specific ground follower (result of Steps (4)–(6)). Example for a sea-ice melt pond (Pond-3775) using the parameters in Table 1 for granule ATL03_20220726163210_05311604_006_02 and beam gt3r (500 m along-track segment length). Figure 3 long description.

Here, we provide a brief overview of the core DDA algorithmic steps, illustrated with example plots at each stage. The examples use a 500 m ICESat-2 sea-ice segment containing a large melt pond (Pond-3775), as shown in Fig. 3.

*(Step 1) Large-scale separation of signal and noise slabs.* Starting with the geolocated photon data in ATL03 (Fig. 3a), a large-scale separation is applied to distinguish noise from signal photons. The full geophysical signal of interest is contained within the ‘signal slab’ (green photons, Fig. 3b), while the ‘noise slab’ (red photons, Fig. 3b) directly above it contains only noise photons. The density characteristics of the noise slab are used to filter out noise from the signal slab. The heights of the slabs are defined by an algorithmic parameter, slab-height (l), which is set to 30 m for all runs in this analysis (Table 1).

*(Step 2) Density calculation.* A density value is calculated for each photon using a radial basis function (*rbf*), providing an additional dimension for analysis. Centered on a given photon, the *rbf* is used as a kernel to weight nearby photons based on their distance from the center photon. A 2-dimensional Gaussian function describes the weight distribution of the *rbf*-kernel, with its shape and size defined by three DDA input parameters: (1) sigma (s), the standard deviation of the Gaussian distribution; (2) cutoff (u), the number of standard deviations used in weighting; and (3) anisotropy (a), the factor that skews the shape of the kernel in an anisotropic manner. An anisotropy value greater than 1 increases the weight assigned to neighboring photons in the horizontal direction, which is useful when the expected geophysical reflector is relatively flat, such as an unfractured sea-ice surface. The density field for the example sea-ice melt pond is given in Fig. 3c.

*(Step 3 ) Auto-adaptive threshold function.* A density threshold is used to classify photons within the signal slab in varying noise situations. A threshold value is determined every 5 m along-track, as given by the ‘bin-width for thresholding’ parameter ($t_{bin}$) (see Herzfeld and others (2023)), and consists of two components. The first component adds a small offset value, given by the threshold-bias-offset parameter (k), to the maximum noise density associated with each bin (as given by noise-slab densities). The photons with densities below this value are classified as ‘false signal’ (dark green photons in Fig. 3d), and are conceptualized as noise photons within the signal slab that have similar densities to those in the noise slab.

The second part of the density threshold calculates a quantile of the remaining photon densities, as determined by the threshold-quantile parameter q. Photons with densities above the q-quantile value are classified as ‘post-quantile’ (light green in Fig. 3d), while photons with densities below the quantile are classified as ‘pre-quantile’ (blue photons in Fig. 3d). As a result, we are left with ‘post-quantile’ signal photons, or thresholded photons, as given by the colored photons in Fig. 3e.

#### 3.1.2. Bifurcation steps and melt-pond-depth estimation

The remaining steps are specific to the DDA-bifurcate-seaice algorithm, which estimates surface heights for multiple surfaces within the thresholded signal photons that remain after the thresholding procedure.

*(Step 4) Identification of peaks in along-track photon-height histograms.*

The goal now is to implement a bifurcation criterion to identify regions with one or two surfaces and assign the thresholded signal photons to the appropriate surface. To achieve this, we compute along-track elevation histograms of the thresholded signal photons. Elevation histogram sizes are controlled by parameters z and v (Table 1). Histograms are then smoothed using a binomial filter, and peaks are identified using parameters for height (M) and prominence (O).

In the current analysis, the maximum number of peaks allowed is two, restricting the algorithm to the identification of at most two surfaces. Therefore, based on the identification of peaks in the smoothed elevation histograms, two cases arise: a single surface (indicating no melt pond) or potentially two surfaces (suggesting the presence of a melt pond). This forms the bifurcation criterion. The second case involves only the potential for two surfaces, with false positive melt ponds identified and removed in Step 6 below.

*(Step 5) Depth determination using ground-follower function for melt ponds.*

In the case of a single peak and a single surface, the standard ground-following procedure is applied to interpolate the thresholded signal photons, resulting in a standardized surface at the resolution defined by the parameter R. The surface is binned every R meters along-track, and the height estimate for each bin is determined by taking the mean height of the thresholded photons, weighted by their density. When the unweighted standard deviation of photon heights in the bin exceeds the threshold defined by the parameter S, the ground follower resolution is increased by a factor of r across the bin of width R, and the same height determination is applied over the shorter bin widths. This increase in ground-following resolution is applied to better capture vertical features, such as crevasses (Herzfeld and others, 2021b) or topographic relief of the bottom of a melt pond, which often exhibit significant heterogeneity over short distances.

When two peaks are identified, the thresholded signal photons are divided into top- and bottom-surface sets based on the shape of the histogram around the two peaks (Herzfeld and others, 2023). The top and bottom sets of thresholded photons are then separately passed to the standard ground follower algorithm. The top surface utilizes a density-weighted mean to assign elevation estimates for each along-track bin, while the bottom surface utilizes a density-weighted elevation quantile given by the melt-pond-depth quantile parameter (dq). This is the key parameter controlling melt-pond-depth estimates, which we optimize in the later sections of the analysis using coincident Chiroptera data.

*(Step 6) Correcting melt pond shapes and removing false positives.*

In the final step, each pond is analyzed individually based on its shape to eliminate false positive pond identifications and improve the pond edge location estimates. False positives are removed using three geometric criteria: (1) a minimum width based on the number of depth estimates (mw), (2) a minimum depth (md) and (3) the flatness of the top surface, as determined by the top-surface ground follower. This is based on the expectation that pond surfaces are relatively flat compared to the surrounding ice. If a pond passes all three criteria, the top-surface height estimates for the entire pond are set to the mean top-surface height of all interior and edge points.

The final results of Steps (4)–(6) are the interpolated and corrected surface heights for the top surface (red line) and bottom surface (green line), fitted to the thresholded signal photons (Fig. 3f). In addition, the DDA-bifurcate-seaice provides estimates of individual pond characteristics quantifying the spatiotemporal locations of pond edges, width, mean depth, maximum depth, pond surface height and mean saturation characteristics of the ATLAS receivers.

### 3.2. Comparison to airborne data

#### 3.2.1. Sea-ice drift correction

To accurately align measurements from Chiroptera and ICESat-2, it is necessary to correct for sea-ice drift between their respective acquisition times, which is less than 40 min for FL1, as Arctic drift velocities can reach several kilometers per day (Plotnikov and others, 2024). For the depth analysis of individual ponds in this paper, we apply a manual drift correction in the x and y directions to the ICESat-2 data, based on matching distinct features in both the Chiroptera-515 and ICESat-2 photon distributions, such as ridges and melt-pond edges. This process is aided by the Chiroptera imagery for each pond, where key features are seen from above. Chiroptera imagery and lidar data are collected simultaneously and are therefore already co-geolocated. Chiroptera-515 and drift-corrected ICESat-2 photon distributions, along with associated imagery, are plotted separately and together for each pond in this analysis.

Since melt-pond bottoms are non-uniform in shape and depth, this careful, and sometimes tedious, manual feature matching is required to ensure the lidar transects overlap, allowing comparison of coincident depth measurements. The exact drift correction for each of the 10 ponds in this analysis is given in Table S2 of the supplementary material.

Saylam and others (2025) have developed a sea-ice drift correction method called the LidarShift Algorithm and have applied it to their analysis of the 2022 NASA ICESat-2 Project Arctic Summer Sea Ice Campaign data. Although this algorithm has been used to correct drift in corresponding ICESat-2 data from FL1 and FL2 of the 26 July 2022 flight using 3 km segments, we opted for a manual correction approach. This decision was based on the smaller dataset analyzed and our goal of achieving the most precise alignment between ICESat-2 and Chiroptera-515 data for each individual pond. That is, our goal is to apply drift corrections at spatial scales comparable to individual melt ponds (≤200 m), rather than over coarser 3 km segments. Our manual drift corrections are consistent with the bulk estimates provided by Saylam and others (2025) in both magnitude and direction.

#### 3.2.2. Melt pond depths from Chiroptera-515 data

Surface height estimation using Chiroptera-515 takes as input the geolocated photon data (x,y,z,t), which is pulled from its native laz-formatted data files (Saylam and others, 2025). Since the swath-width of the Chiroptera-515 data during this flight segment extends roughly 500 m in the direction perpendicular to RGT 531, the data is down-sampled to a radius of 2 m around the drift-corrected ICESat-2 RGT line. This enables analysis in the 2-D plane of along-track distance and elevation, as utilized in the DDA-bifurcate-seaice analyses and visualizations.

The Chiroptera-515 top surface is determined by fitting a horizontal line to the top-most photons between visually determined pond edges in the photon cloud. Bottom surface heights from Chiroptera are provided by a density-weighted calculation of Chiroptera-515 photons below the flat pond top-surface returns, as described for ICESat-2/DDA data in Step 5 in Section 3.1.2.

In addition to drift correction, the depth-comparison analysis requires adjustment of the DDA-bifurcate-seaice melt-pond top-surface height to align with the corresponding Chiroptera-515 top-surface height for each individual pond. The height correction reduces the DDA-bifurcate-seaice top-surface heights by approximately 17 m. The need for this correction is mostly attributable to parameters used for modeling the geoid, and to a lesser extent, correcting for tide and inverse-barometer effects in the ATL03 data product and should be considered a local correction. Exact height corrections for each pond in this analysis are found in Table S2.

### 3.3. Optimization of the melt-pond-depth quantile in the DDA-bifurcate-seaice

This study focuses on optimizing the qd parameter, which governs melt-pond-depth estimation. To achieve this, we compare Chiroptera-515 and DDA-bifurcate-seaice depth estimates for ten large melt ponds (width >30 m) identified in the ≈125 km FL1 flight segment. A Chiroptera RGB image is provided for each pond to aid in the comparative analysis. We present an analysis of 10 melt ponds that illustrate the DDA-bifurcate-seaice functionality across different pond characteristics such as pond depths, pond bottom morphologies and a range of photon cloud characteristics.

These ponds are some of the few to meet both the minimum width and minimum depth requirements as prescribed by the algorithmic parameters (see Table 1 and Section 3.1.2). Moreover, the selected ponds are free of any saturation effects, which are common in ponds detected in FL2, and are usually associated with an ICESat-2 transect that bisects the center of a pond. If the transect is along the edge of a pond, photons may be returned equally from the pond and the ice edge within the ICESat-2 footprint, which can complicate melt-pond identification and depth estimation. Of note, ICESat-2 data in this study were acquired during daytime conditions, which present a greater detection challenge due to the elevated background noise characteristic of ICESat-2 daytime observations. While many more smaller (<30 m width) and shallower (<0.5 m maximum depth) ponds exist along FL1, as seen in the imagery, they do not provide distinct enough signals in the photon data for the DDA-bifurcate-seaice to detect confidently in its current stage of development.

**Figure 4.**
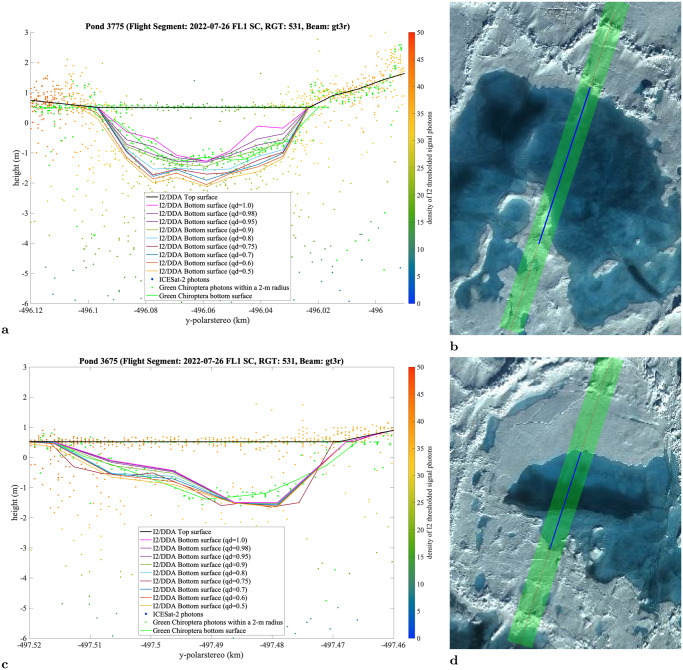
DDA-bifurcate-seaice bottom surface estimates for varying qd values compared to estimates from Chiroptera-515 data. Two large ponds surveyed on 26 July 2022, Chiroptera data-swath 1 (FL1). Pond depths for varying qd parameters and for Chiroptera data (green dots/line) for (a) an ≈80 m pond (id=3775) and (c) a ≈50 m pond (id=3675). (b) Pond-3775 and (d) Pond-3675 in Chiroptera RGB imagery with the ICESat-2 survey path (red line) across the pond width (blue line) and the approximate 11 m diameter footprint of ICESat-2 (transparent green line). Figure 4 long description.

The optimization procedure, carried out for each individual pond, begins by running the DDA-bifurcate-seaice for a range qd-values, which yields unique bottom surface height estimates for each value (given by the multi-colored lines in Fig. 4). Specifically, the DDA-bifurcate-seaice is run with qd-values in the set

$$\begin{array}{cc} qd & amp;\in [0.0,0.1,0.2,0.3,0.4,0.5,0.6,0.7,\\ & amp;\;0.75,0.8,0.9,0.95,0.98,1.0] \end{array}$$

Note that qd=0.75 was the previously used default value in depth determination (Herzfeld and others, 2023).

The along-track resolutions of the Chiroptera and DDA-bifurcate-seaice height estimates are identical, though their exact estimation locations may be slightly offset. To enable direct comparison, the ICESat-2/DDA bottom surface heights are interpolated to the along-track locations of the Chiroptera-515 estimates for each run. The accuracy of the interpolated ICESat-2/DDA heights is evaluated with respect to the Chiroptera-515 estimates (green lines in Fig. 4) using a Mean Squared Error (MSE) metric across each pond transect. The MSE quantifies the average squared difference between the interpolated DDA-bifurcate-seaice bottom heights ($h_{DDA})$ and the corresponding Chiroptera-515 bottom heights ($h_{chir}$) across each pond. For a given pond p, and melt-pond-depth quantile qd, the squared-difference measure, $MSE_{p,qd}$, for a particular run is given by

(1) $$MSE_{p,qd}=\frac{1}{n}\sum_{i=1}^{n}(h_{DDA,i}-h_{chir,i}{)}^{2}$$

for all estimation locations i=1,...,n across the pond transect. A mean is used to facilitate the comparison of depth differences between the ten ponds, which vary in width and the number of along-track depth estimates. MSE measures for all runs in the melt-pond-depth optimization can be found in the supplementary material (Table S1). The final result is an optimal qd value, ($qd=qd_{opt}$), for each pond corresponding to the minimum MSE measure.

## 4. Results

### 4.1. Individual ponds

**Figure 5.**
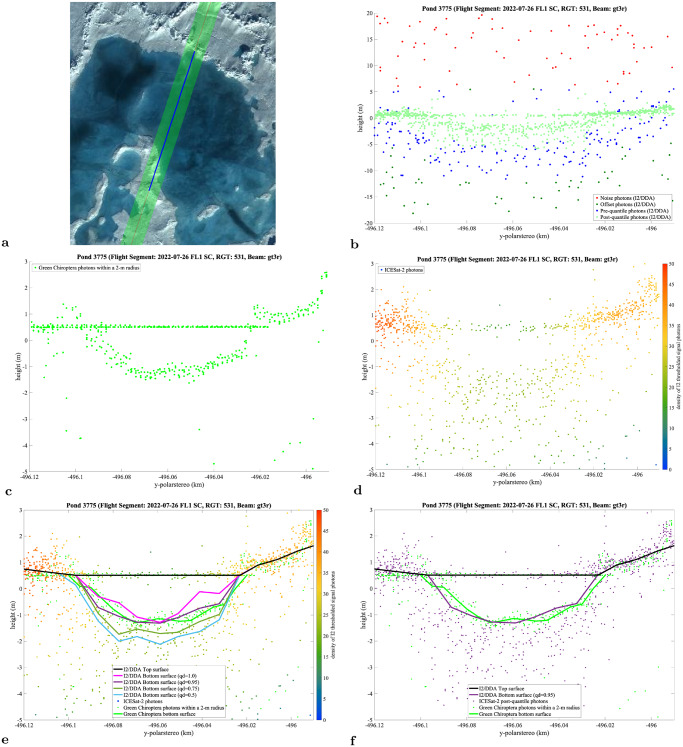
DDA-bifurcate-seaice and Chiroptera-515 photon distributions and surface heights for various depths over Pond-3775. (a) Pond-3775 in Chiroptera imagery with survey path across the pond given by the red line, the DDA-determined pond width by the blue line, and the extent of the 11 m footprint of ICESat-2 in green. (b) ICESat-2/DDA photon classification based after the thresholding procedure. (c) Chiroptera-515 photons within a 2 m radius of ICESat-2 survey line. (d) ICESat-2 photons weighted by density. (e) Pond depths with various depth quantiles (qd) with the Chiroptera-515 bottom surface estimate (green line). (f) Optimal depth given by qd=0.95 (purple line). Figure 5 long description.

**Figure 6.**
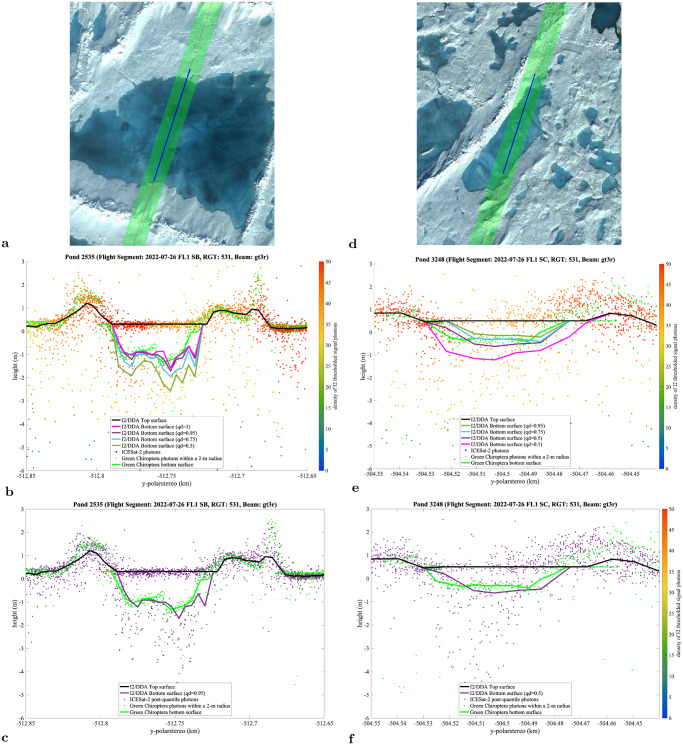
DDA-bifurcate-seaice and Chiroptera-515 photon distributions and surface heights for various depths over (a)–(c) Pond-2535 and (d)–(f) Pond-3248. The full plot sequences for both Pond-2535 and Pond-3248 are found in the supplement (Figs. S1 and S2, respectively). (a) Pond-2535 and (d) Pond-3248 in Chiroptera imagery with survey path across the pond given by the red line, the DDA-determined pond width by the blue line, and the extent of the 11 m footprint of ICESat-2 in green. (b) Pond-2535 and (e) Pond-3248 depths with various depth quantiles (qd) with the Chiroptera-515 bottom surface estimate (green line). (c) Pond-2535 and (f) Pond-3248 optimal depth given by qd=0.95 (purple line). Figure 6 long description.

**Figure 7.**
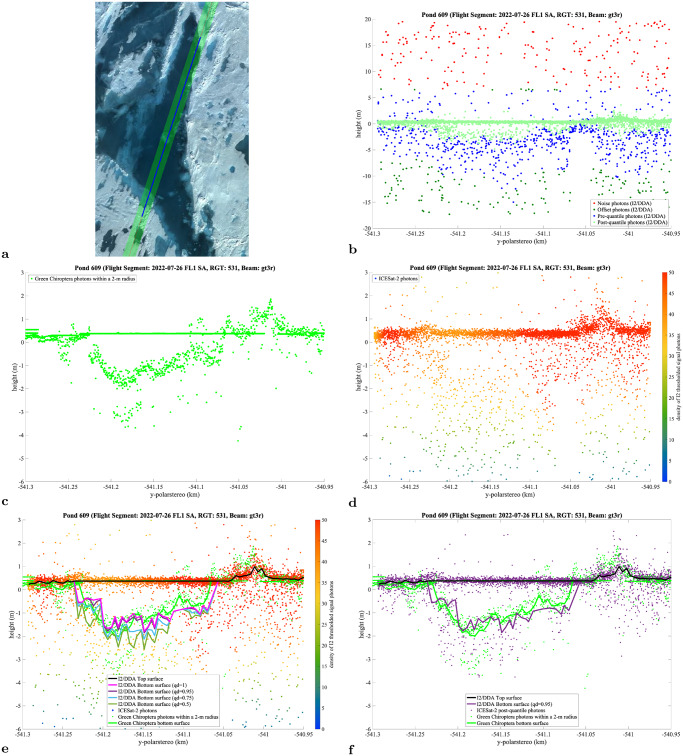
DDA-bifurcate-seaice and Chiroptera-515 photon distributions and surface heights for various depths over Pond-609. (a) Pond-609 in Chiroptera imagery with survey path across the pond given by the red line, the DDA-determined pond width by the blue line, and the extent of the 11 m footprint of ICESat-2 in green. (b) ICESat-2/DDA photon classification based after the thresholding procedure. (c) Chiroptera-515 photons within a 2 m radius of ICESat-2 survey line. (d) ICESat-2 photons weighted by density. (e) Pond depths with various depth quantiles (qd) with the Chiroptera-515 bottom surface estimate (green line). (f) Optimal depth given by qd=0.95 (purple line). Figure 7 long description.

**Figure 8.**
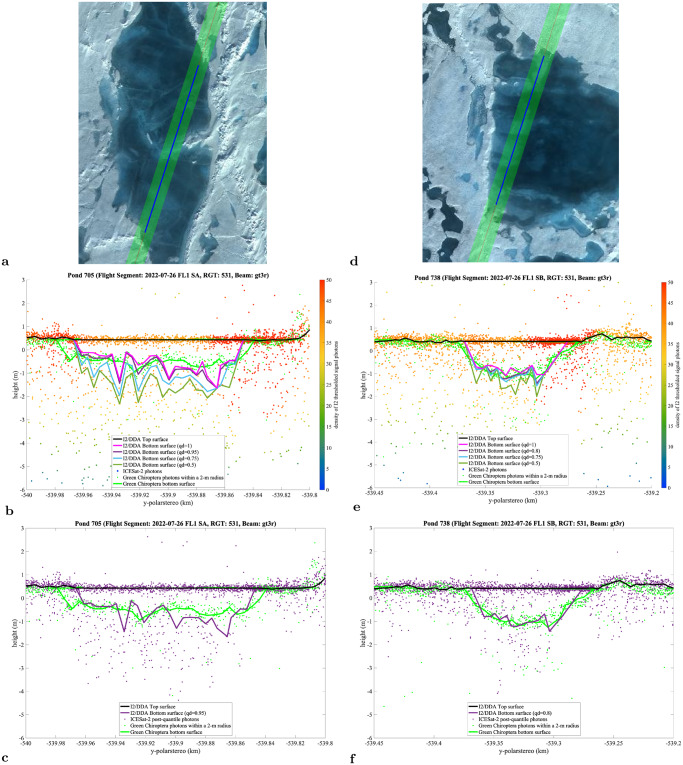
DDA-bifurcate-seaice and Chiroptera-515 photon distributions and surface heights for various depths over (a)–(c) Pond-705 and (d)–(f) Pond-738. The full plot sequences for both Pond-705 and Pond-738 are found in the supplement (Figs. S3 and S4, respectively). (a) Pond-705 and (d) Pond-738 in Chiroptera imagery with survey path across the pond given by the red line, the DDA-determined pond width by the blue line, and the extent of the 11 m footprint of ICESat-2 in green. (b) Pond-705 and (e) Pond-738 depths with various depth quantiles (qd) with the Chiroptera-515 bottom surface estimate (green line). (c) Pond-705 and (f) Pond-738 optimal depths given by qd=0.95 and qd=0.8, respectively (purple lines). Figure 8 long description.

**Figure 9.**
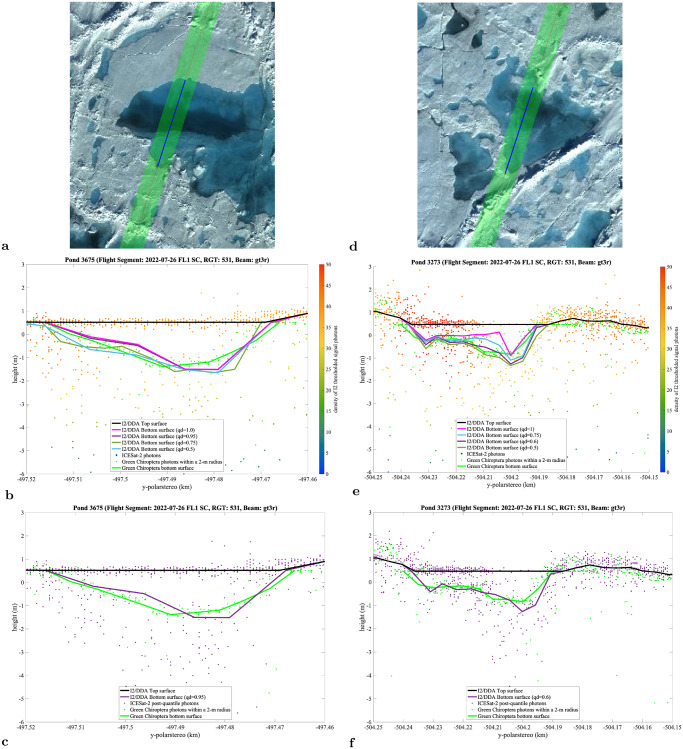
DDA-bifurcate-seaice and Chiroptera-515 photon distributions and surface heights for various depths over (a)–(c) Pond-3675 and (d)–(f) Pond-3273. The full plot sequences for both Pond-3675 and Pond-3273 are found in the supplement (Figs. S5 and S6, respectively). (a) Pond-3675 and (d) Pond-3273 in Chiroptera imagery with survey path across the pond given by the red line, the DDA-determined pond width by the blue line, and the extent of the 11 m footprint of ICESat-2 in green. (b) Pond-3675 and (e) Pond-3273 depths with various depth quantiles (qd) with the Chiroptera-515 bottom surface estimate (green line). (c) Pond-3675 and (f) Pond-3273 optimal depths given by qd=0.95 and qd=0.6, respectively (purple lines). Figure 9 long description.

Figures 5–9, along with Figs. S1–S8 in the supplement, illustrate depth comparisons between Chiroptera-515 and ICESat-2/DDA measurements for each of the ten analyzed melt ponds. Complete plot sequences, found here (Figs. 5 and 7) and in the supplement (Figs. S1–S8), focus on a specific pond and include Chiroptera imagery with survey lines (subfigures **a**), DDA-bifurcate-seaice photon classifications (subfigures **b**), photon distributions from both Chiroptera-515 (subfigures **c**) and drift-corrected ATLAS/ICESat-2 (subfigures **d**) measurements, DDA-bifurcate-seaice depth estimates across a range of qd values (subfigures **e**), and a comparison of depth estimates at $qd=qd_{opt}$ with the Chiroptera-515 estimates (subfigures **f**). Abbreviated pond-example plots (Figs. 6, 8 and 9) include only the key subfigures **a**, **e** and **f**.

Table 2 summarizes the results of our analysis, providing a unique pond identification number, pond location in the ICESat-2 ATL03 photon data given by the delta_time variable, pond surface heights, maximum depths and $qd_{opt}$ values for each pond. Two maximum depth estimates are provided for each pond using both a fixed qd=0.75 value and for each pond’s corresponding $qd_{opt}$ value. Notably, each melt-pond surface height is above the sea-level height estimate of 0.222 m given by Chiroptera-515 data for FL1, ranging from 0.129 m (Pond-4108) to 0.295 m (Pond-3675) above sea-level.

**Table 2.** Depth quantile parameter optimization results for the ten characteristic sea-ice melt ponds from the FL1 segment of the 26 July 2022 campaign flight. Each pond is given a unique ID and has associated plots given by the figure number(s) (Fig. #). The estimated sea-level height across FL1 is 0.222 m. Ponds ordered by their associated ICESat-2 delta_time value. ICESat-2 delta_time values correspond to the measurement time in the ATL03_20220726163210_05311604_006_02 granule for beam gt3r corresponding to the near the center of the pond. Table 2 long description.

**Pond ID**	**Fig. #**	**Center** delta_time **(s)**	**Flight segment**	**Surface height (m)**	**Max. depth (m)** qd=0.75	**Max. depth (m)** $qd=qd_{opt}$	**Optimal depth quantile**
4311	S7	144088549.159566	FL1-SC	0.418	0.906	1.318	0.4
4108	S8	144088549.501658	FL1-SC	0.351	0.977	1.265	0.5
3775	5	144088550.279792	FL1-SC	0.503	2.400	1.855	0.95
3675	9,S5	144088550.504517	FL1-SC	0.510	2.180	2.034	0.95
3273	9,S6	144088551.564643	FL1-SC	0.467	1.563	1.730	0.6
3248	6,S2	144088551.607295	FL1-SC	0.502	0.912	1.124	0.5
2535	6,S1	144088552.905006	FL1-SB	0.309	2.259	2.020	0.98
738	8,S4	144088557.049109	FL1-SB	0.403	1.953	1.840	0.8
705	8,S3	144088557.139189	FL1-SA	0.403	2.393	2.073	1.0
609	7	144088557.332093	FL1-SA	0.374	2.550	2.223	0.95

#### 4.1.1. Clear ponds with smooth bottom surfaces

Pond-3775, located along FL1-SC, appears to consist of only clear sea-ice meltwater, without intrusion of darker lead water, that is, sea water, across the ≈80 m transect (Fig. 5). It has a well-defined photon distribution with minimal volume scattering within the water column and a smooth U-shaped bottom surface. Using a melt-pond-depth quantile of qd=0.75, Pond-3775’s maximum depth is 2.400 m; however, using a qd value of 0.95 best matches the corresponding Chiroptera-515 bottom surface (see Fig. 5f). With $qd_{opt}=0.95$, the maximum depth estimate is lowered to 1.855 m.

Large ponds consisting of pure meltwater, like Pond-3775, and also Pond-2535 (Fig. 6a–c and Fig. S1 in the supplement), are easiest for the DDA-bifurcate-seaice to detect without significant sensitivity to algorithmic parameters. The non-uniform, crescent-like shape of Pond-3775, however, complicates the width estimate due to the large dependence on the transect location across the pond. For example, if the ICESat-2 on-ice beam center was shifted to the right 20 m, the width estimate would be approaching 100 m, while a 20 m shift to the left would yield a width estimate of 60 m. Thus, pond-edge estimation is complicated as ICESat-2’s on-ice footprint diameter is ≈11 m (green outline in Fig. 5a), and the along-track photon distribution contains a range of pond-edge photons around the ‘true’ pond-edge location at the beam’s center (blue line in Fig. 5a).

Pond-3248 (Fig. 5d–f and Fig. S2) provides another example of a clear melt-pond with a smooth bottom consisting of only meltwater. While still detected by the DDA-bifurcate-seaice, its characterization is more difficult than that of the larger Pond-3775 and Pond-2535 due to its shallow meltwater depths. The algorithm is still able to provide pond width and depth estimates for Pond-3248 despite additional complications due to its oblong shape and immediate proximity to ridged ice along one of its longer sides.

#### 4.1.2. Dark ponds with rough bottom topography

The RGB image of Pond-609 shows a dark water color, which arises from sea-water intrusion into the melt pond from below. However, there is still a distinct pond-bottom as seen in the Chiroptera-515 and ICESat-2 photon distributions (Fig. 7). The longest transect analyzed, ICESat-2, bisects 200 m of Pond-609 across its near-full length. A depth quantile of qd=0.75 yields a maximum depth of 2.55 m, while the optimal depth quantile of $qd_{opt}=0.95$ reduces the maximum depth estimate to 2.223 m.

Connectivity of melt ponds to the underlying seawater predominately occurs from percolation through connected pore structures (porosity) at the pond bottom or through macroscopic cracks or leads (flaws) (Perovich and Polashenski, 2012). As seen in the ice surface topography estimates given by the solid lines in Fig. 7e–f, Pond-609 has a very rough pond bottom across its width, with height estimates varying up to 0.5 m between consecutive along-track estimates. The rough bottom topography may be an indication of the presence of macroscopic flaws that allow sea-water intrusion. Notably, the minimum MSE measure for this pond was the largest across all 10 ponds analyzed (Table S1), indicating the poorest match between ICESat-2 and Chiroptera-515 pond bottom height estimates, which is likely a reflection of its structural and/or material properties.

The rough bottom surface topography detected by the DDA-bifurcate-seaice is not entirely an artifact of sparse signal photons, but indeed captures the complex depth profile of the pond. This is more apparent in Pond-705 that also has a rough bottom topography (Fig. 8a–c and Fig. S3), which is visible in the imagery because it is comprised of more clear meltwater, and less dark sea water, than Pond-609. Rough bottom topography is also apparent in the Chiroptera-515 data, though height variability across the transect is less severe likely due to relative signal strength. The roughness of the bottom surface, and the macroscopic cracks that may be present, is due mostly to deformation from sea-ice dynamics rather than causes arising from particular drainage characteristics.

The ICESat-2 transect across Pond-705 is 135 m long, and its depth at qd=0.75 is 2.393 m. The optimal depth quantile for this pond is given by $qd_{opt}=1.0$, though the MSE measure for qd=0.98 and qd=0.95 are very close (Table S1), indicating near-optimal depth matches at each of these melt-pond quantile values. Using the optimal depth quantile, the maximum depth estimate is reduced to 2.073 m.

Pond-738 is also a darker pond with rough bottom topography; however, the ICESat-2 transect bisects the pond near its shallower edge, where the pond bottom is visible in the imagery (Fig. 8d–f and Fig. S4). Clear topographical features oriented approximately perpendicular to the ICESat-2 survey line are visible in the imagery. Based on their location and spacing, these features correspond to noticeable bumps in the bottom ice surface estimate provided by ICESat-2/DDA (Fig. 8e–f), though estimates from runs using lower depth quantiles, for example, qd=0.5, appear to better capture the spatial variability in height across the transect.

The length of the Pond-738 transect is 105 m, and with a depth quantile of qd=0.75, the maximum depth estimate is 1.953 m. The optimal depth quantile is close to this estimate at $qd_{opt}=0.8$, which adjusts the maximum depth estimate to 1.840 m.

We find no significant correlation between pond-bottom roughness and the optimal melt-pond-depth quantile, nor between roughness and the difference in depth estimates derived from qd=0.75 and $qd=qd_{opt}$. However, given the limited sample size, these results are insufficient to draw definitive conclusions about the influence of bottom roughness on the optimized depth estimate. Among the ten ponds, Ponds 609 and 708 show the largest depth deviations from the coincident Chiroptera-515 data, whereas Pond-738 shows the smallest (see Table S1). Therefore, there is no indication that pond-bottom roughness significantly impacts the depth retrieval of DDA-bifurcate-seaice, as its effects are mitigated by the reduced ground-follower resolution (see Step 5 in Section 3.1.2).

#### 4.1.3. Partially drained ponds

Some ponds appear to have partially drained their meltwater, as identified in the imagery, where a pond is surrounded by smooth and relatively flat ice-surface topography of height similar to the top surface height of the melt pond. The DDA-bifurcate-seaice algorithm is still able to identify pond edges in this situation despite the similarity in the surface returns due to the subsurface (or bottom) returns identified in the bifurcation procedure of the algorithm.

Pond-3675 is one such example of a partially drained pond (Fig. 9a–c and Fig S5). This pond consists mostly of clear and relatively shallow meltwater with noticeable dark sea-water intrusion at its deepest part near the center. The smooth bottom topography, coupled with the dark water located at the deepest point, points to Pond-3675 being connected to the sea water through connected pore structures and a permeable bottom surface rather than macroscopic cracks or flaws as seen in the ponds with rough bottoms (Section 4.1.2).

The ICESat-2 transect across Pond-3675 is approximately 55 m and has a maximum depth of 2.180 m when qd=0.75. The optimal depth quantile of $qd_{opt}=0.95$ reduces the maximum depth estimate to 2.034 m.

Pond-2535, found in the supplement (Fig. S1), also appears to be a partially drained pond, though its connection to the underlying sea water is less apparent. While the pond surface height of Pond-2535 is the lowest of all ponds at 0.309 m, Pond-3675 has the highest surface height of 0.517 m. This implies that pond-surface height may not be an indicator of the connectivity to sea water and the amount of meltwater drained, but is instead related to the pre-melt sea-ice topography (Perovich and Polashenski, 2012).

Pond-3273 is a small, shallow pond composed primarily of clear meltwater, with darker water visible at its deepest point (Fig. 9d–f and Fig. S6), a characteristic also observed in Pond-3675. This feature suggests high permeability of the pond bottom at the deepest location, indicating a connection to underlying seawater. However, no clear evidence of partial drainage is apparent in the imagery. This absence of drainage may be attributed to the low meltwater volume in Pond-3273 and the resulting small hydraulic head, which is insufficient to drive drainage into the ocean (Perovich and Polashenski, 2012).

Pond-3273 has an ICESat-2 transect length of 55 m across its full width and an estimated maximum depth of 1.563 m when using qd=0.75. The optimal melt-pond-depth quantile for this pond is $qd_{opt}=0.6$, which increases the maximum depth estimate to 1.730 m.

Across all analyzed ponds, there is no clear correlation between melt-pond depth and the depth discrepancies between the DDA-bifurcate-sea-ice retrievals and the coincident Chiroptera-515 measurements (see Table S1). More broadly, no consistent relationship is observed between depth discrepancies and other melt-pond characteristics, such as surface roughness or the darkness of the pond water. While additional coincident validation data are not presently available, future airborne acquisitions may enable verification of these findings across a wider range of pond conditions.

### 4.2. Improvement of depth determination using optimized depth-quantile values

We now quantify how using the discretely optimized melt-pond-depth quantiles, $qd_{opt}$, improves depth estimation accuracy and reduces associated uncertainty compared to using a global value of qd=0.75. The use of pond-specific, or *discretely* optimized, melt-pond-depth quantiles ($qd_{opt}$) represents a best-case scenario for minimizing error, quantified by MSE measures, under the current algorithmic framework described in Section 3.1.2.

Table 3 provides the bias and MSE for DDA-bifurcate-seaice depth estimates across 10 melt ponds, when using the two melt-pond-depth quantile options. MSE is defined in Eqn (1), and the corresponding values for all runs included in this analysis are presented in Table S1 of the supplement. The bias in depth estimation, $bias_{p,qd}$ for a given pond p, and melt-pond-depth quantile qd, is given by

(2) $$bias_{p,qd}=\frac{1}{n}\sum_{i=1}^{n}(h_{DDA,i}-h_{chir,i})$$

where $h_{DDA,i}$ and $h_{chir,i}$ are the heights of the bottom surface provided by the DDA-bifurcate-seaice and Chiroptera, respectively.

**Table 3.** Depth determination differences when using qd=0.75 and the optimized $qd=qd_{opt}$ melt-pond-depth quantile parameter. The optimal melt-pond-depth quantile ($qd_{opt}$), identified through the analysis in Section 4.1, appears in Column 2. Columns 3 and 4 report the bias (Eqn (2)) and MSE (Eqn (1)) of depth differences between Chiroptera and the DDA-bifurcate-seaice estimates, computed using a fixed qd=0.75 and the pond-specific $qd=qd_{opt}$ values, respectively. Column 5 shows the difference in maximum depth estimates, and Column 6 provides the difference in mean depth estimates between the two depth-quantile assignments. Column 7 presents the maximum absolute pointwise depth difference between the two depth-quantile assignments, calculated across all along-track estimation points within each pond. Table 3 long description.

		**Bias / MSE**	**Bias / MSE**			
**Pond ID**	$qd_{opt}$	qd=0.75 **(m/m**${}^{2}$**)**	$qd=qd^{*}$ **(m/m**${}^{2}$**)**	Δ **max-depth (m)**	Δ **mean-depth (m)**	max(|Δdepth|) **(m)**
4311	0.4	−0.29 / 0.12	−0.074 / 0.043	−0.4114	−0.2396	0.4565
4108	0.5	−0.12 / 0.12	0.11 / 0.042	−0.2878	−0.10799	0.4404
3775	0.95	0.35 / 0.27	0.0017 / 0.080	0.5307	0.4265	0.7875
3675	0.95	0.13 / 0.16	0.0053 / 0.086	0.05314	0.1573	0.3726
3273	0.6	−0.11 / 0.082	0.027 / 0.062	−0.1675	−0.08057	0.3601
3248	0.5	0.045 / 0.21	0.19 / 0.16	−0.2111	0.2085	0.3089
2535	0.98	0.27 / 0.018	0.11 / 0.10	0.3180	0.3261	0.6505
738	0.8	−0.025 / 0.38	−0.055 / 0.037	0.1130	0.04577	0.1579
705	1.0	0.34 / 0.41	−0.065 / 0.17	0.3204	0.5218	1.114
609	0.95	0.24 / 0.22	0.069 / 0.18	0.3273	0.2252	0.6413
**Avg.**	**0.763**	**0.083 / 0.22**	**0.032 / 0.096**	**0.05848**	**0.1066**	**0.5478**

Additionally, Table 3 reports differences in key melt-pond characteristics, including maximum depth, mean depth and the maximum absolute depth difference. The latter is defined as the greatest pointwise depth discrepancy between estimates at individual along-track locations within each pond.

The use of discretely optimized melt-pond-depth quantiles improves average depth-bias estimates by a factor of 2.6, reducing the bias for deeper pond estimates to 0.032 m from 0.083 m when using qd=0.75 for all ponds. In addition, the MSE has improved on average from 0.22 m${}^{2}$ to 0.096 m${}^{2}$, representing an approximate 2.3-fold reduction in error. These results demonstrate the potential for improved depth estimation through refinement of the melt-pond-depth quantile parameter within the DDA-bifurcate-seaice framework.

In the context of pond characterization, the use of pond-specific $qd_{opt}$ values reduces the average mean-depth estimate across the analyzed ponds by 0.1066 m. Estimates of maximum pond depth also decrease, with an average reduction of 0.05848 m. These findings indicate that imposing a global qd=0.75 leads to a systematic overestimation of both mean melt-pond depth and, by extension, total melt volume when scaled. This consideration is particularly important when interpreting results from the DDA-bifurcate-seaice experimental data product in release 7 of ATL07.

### 4.3. Optimal melt-pond-depth quantile and its relation to mean depth

Building on the discretely optimized melt-pond-depth quantile analysis presented in the previous section, we now aim to develop a practical method for assigning melt-pond quantiles within the DDA-bifurcate-seaice framework. Specifically, we seek to establish a functional relationship between maximum pond depth and the optimized depth quantile, which will enable the replacement of the currently used global melt-pond-depth quantile value of 0.75 in future releases.

We begin by examining the correlation between the maximum pond depth obtained using qd=0.75 and the corresponding optimized quantile value, $qd_{opt}$. In general, shallow ponds in this analysis are those with maximum depths near or below 1.5 m, while deeper ponds have depths closer to and exceeding 2 m. We find that deeper ponds will have larger $qd_{opt}$-values (typically 0.95) while shallower ponds have smaller $qd_{opt}$ values (0.4-0.6). This implies that previous depth estimates that used the default depth quantile value of qd=0.75 need to be adjusted shallower for deeper ponds, and vice versa, with larger qd values providing shallower depth estimates.

**Figure 10.**
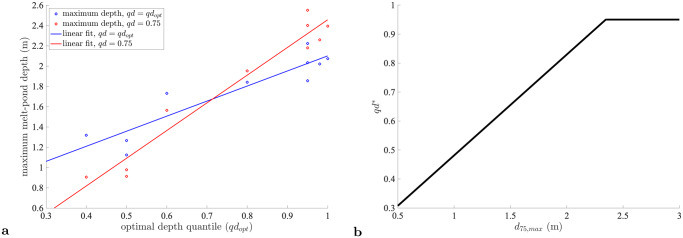
Relationship between melt-pond quantile parameter values qd and maximal depths. (a) Maximum melt-pond depths for optimal depth quantiles ($qd=qd_{opt}$, blue) and for the 0.75 quantile (qd=0.75, red). (b) The depth-quantile function describing the relationship between improved depth quantile value in the updated algorithm, $qd^{*}$, given by the maximum depth when using qd=0.75, $d_{75,max}$ (see Eqn (3)). Figure 10 long description.

Figure 10a shows the maximum depths of each pond for both qd=0.75 (red) and $qd=qd_{opt}$ (blue), with a linear fit applied to each dataset. A clear relationship emerges between a pond’s maximum depth and its corresponding optimal depth parameter ($qd_{opt}$), which can be approximated using the linear fit. Notably, in deeper melt ponds (maximum depth > 1.7 m), the pond bottom is closer to the top of the signal-photon cloud, whereas in shallower ponds, the bottom more closely aligns with the mean height of the signal-photon cloud.

Using this linear relationship, we derive the depth-quantile function (Eqn (3)) to include as an algorithmic refinement to the DDA-bifurcate-seaice for depth estimation. Determination of the melt-pond-depth quantile in the algorithm now goes as follows. First, the maximum depth of each pond is determined using the previous default melt-pond-depth quantile of qd=0.75. Next, based on the maximum depth value, $d_{75,max}$, an improved melt-pond-depth quantile, $qd=qd^{*}$, is found using Eqn (3), and bottom surface height estimates are recalculated.

The depth-quantile function is plotted in Fig. 10b, and is given mathematically as:

(3) $$qd^{*}=\left\{ \begin{array}{cc} 0.348\cdot d_{75,max}+0.132, & amp;\text{if }d_{75,max}\leq 2.35\;m\\ 0.95, & amp;\text{if }d_{75,max}>2.35\;m \end{array}\right.$$

Notably, the $d_{75,max}$ has a lower bound equal to the minimum-depth parameter, currently set at md=0.5 m (Table 1), resulting in a minimum possible $qd^{*}$ value of 0.3074. Additionally, a depth-quantile above 0.95 is never assigned, as values very close to 1.0 are overly sensitive to outlier photons in the bottom surface signal-photon cloud. Consequently, any pond with a $d_{75,max}$ value exceeding 2.35 m will be assigned a $qd^{*}$ value of 0.95 in the updated bottom-surface ground-follower function.

### 4.4. Improvement of depth determination using the depth-quantile function

Here, we assess how the depth-quantile function improves depth estimation accuracy and reduces associated uncertainty relative to the use of a fixed global value of qd=0.75. In the original approach, a constant quantile of 0.75 is applied uniformly across all ponds, whereas the updated method dynamically assigns qd as $qd^{*}$ using the depth-quantile function defined in Eqn (3), following the iterative procedure outlined in Section 4.3. These results are also compared to those from the pond-specific discrete optimizations described in Section 4.2. While the depth-quantile function provides a practical enhancement to melt-pond parameterization within the DDA-bifurcate-seaice framework, the discrete optimization approach serves as a benchmark representing the best-case performance.

Table 4 reports the bias and MSE for DDA-bifurcate-seaice depth estimates across 10 melt ponds, comparing results obtained prior to and following the proposed algorithmic update. The table also reports resulting differences in melt-pond maximum depth, melt-pond mean depth and the maximum absolute depth difference.

**Table 4.** Depth determination differences when using the global (qd=0.75) and the improved ($qd=qd^{*}$) melt-pond-depth quantile parameter. The improved melt-pond-depth quantile ($q^{*}$), derived using Eqn (3), is listed in Column 2, while the optimal quantile ($qd_{opt}$), identified through the analysis in Section 4.1, appears in Column 3. Columns 4 and 5 report the bias (Eqn (2)) and MSE (Eqn (1)) of depth differences between Chiroptera and the DDA-bifurcate-seaice estimates, computed using a fixed qd=0.75 and the adaptive $qd=q^{*}$, respectively. Column 6 shows the difference in maximum depth estimates, and Column 7 provides the difference in mean depth estimates between the two depth-quantile assignments. Column 8 presents the maximum absolute pointwise depth difference between the two depth-quantile assignments, calculated across all along-track estimation points within each pond. Table 4 long description.

			**Bias / MSE**	**Bias / MSE**			
**Pond ID**	$qd^{*}$	$qd_{opt}$	qd=0.75 **(m/m**${}^{2}$**)**	$qd=qd^{*}$ **(m/m**${}^{2}$**)**	Δ **max-depth (m)**	Δ **mean-depth (m)**	max(|Δdepth|) **(m)**
4311	0.4473	0.4	−0.29 / 0.12	−0.13 / 0.056	−0.3416	−0.1978	0.3979
4108	0.4720	0.5	−0.12 / 0.12	0.13 / 0.047	−0.3281	−0.1329	0.4892
3775	0.95	0.95	0.35 / 0.27	0.0017 / 0.080	0.5307	0.4265	0.7875
3675	0.8906	0.95	0.13 / 0.16	0.0059 / 0.095	0.03736	0.1079	0.2541
3273	0.6759	0.6	−0.11 / 0.082	−0.044 / 0.065	−0.04264	0.003905	0.2307
3248	0.4494	0.5	0.045 / 0.21	0.24 / 0.16	−0.3034	−0.2742	0.4013
2535	0.9181	0.98	0.27 / 0.23	0.11 / 0.12	0.1544	0.2017	0.4009
738	0.8116	0.8	−0.025 / 0.38	−0.063 / 0.037	0.1357	0.05843	0.1945
705	0.95	1.0	0.34 / 0.41	0.023 / 0.19	0.3198	0.4059	0.9622
609	0.95	0.95	0.24 / 0.22	0.069 / 0.18	0.3273	0.2252	0.6413
**Avg.**	**0.751**	**0.763**	**0.083 / 0.22**	**0.030 / 0.10**	**0.04896**	**0.08247**	**0.4760**

The average improved melt-pond-depth quantile parameter ($q^{*}$) across the 10 ponds is 0.751, indicating that the use of a global value of qd=0.75, as adopted in the release 7 experimental data product on ATL07, is a near-optimal choice when applying a uniform parameter prescription. Additionally, the mean optimal quantile ($qd_{opt}$), derived independently for each pond, is 0.763. This further supports the conclusion that qd=0.75 provides a robust and well-justified global parameter value.

However, implementation of the depth-quantile function offers significant improvement to melt-pond-depth estimation. While bias and MSE values are relatively low for both methods, the bias is reduced from 0.083 m to 0.030 m, and the MSE decreases from 0.22 m${}^{2}$ to 0.10 m${}^{2}$. Although DDA-bifurcate-seaice estimates remain slightly deeper on average than those from Chiroptera-515, the bias has been reduced by approximately a factor of 2.75 through dynamic assignment of qd using the depth-quantile function. Similarly, the MSE improvement by a factor of approximately 2.2 suggests a substantially better fit to the underlying pond-bottom topography.

For qd=0.75, the MSE of 0.22 m${}^{2}$ corresponds to a root mean squared error (RMSE) of approximately 0.47 m, indicating that the typical deviation in estimated pond depth is just under half a meter. With the depth-quantile function applied, the RMSE is reduced to around 0.32 m. These error estimates are within the spread of the most-dense photon height estimates given by Chiroptera-515 surrounding the interpolated depth estimates, as seen in Figs. 5–9. These reductions in bias and MSE demonstrate that the approach utilizing the depth-quantile function yields more accurate and reliable depth estimates, thereby decreasing the uncertainty associated with depth and pond-bottom characterization.

Compared to the $qd_{opt}$ results in Table 3, the depth-quantile function actually yields a slightly lower average depth bias (0.030 m vs 0.032 m). While the average MSE is marginally lower when using the discretely optimized quantile values (0.096 m${}^{2}$ vs 0.100 m${}^{2}$), as expected due to direct MSE minimization during optimization, the difference is small (0.004 m${}^{2}$) relative to the MSE associated with the global qd=0.75 value, which is substantially higher at 0.22 m${}^{2}$. These results suggest that the depth-quantile function retains most of the performance improvement achieved by the discretely optimized approach, offering a practical and effective parameter determination method for future implementations.

For pond characterization, the depth-quantile function reduces average mean-depth estimates by 0.08247 m and maximum depth estimates by 0.04896 m across the analyzed ponds. These bulk difference estimates are comparable to those obtained using the discretely optimized quantile values (Table 3), although they are slightly smaller in magnitude.

Finally, analysis of the maximum along-track depth differences reveals that ponds assigned an improved depth quantile of $q^{*}=0.95$ exhibit the largest pointwise height discrepancies (max(|Δdepth|)) relative to their qd=0.75 counterparts. Additionally, as shown in Table 3, the largest pointwise depth difference by far is associated with Pond-705, which uses an optimized quantile value of 1.0. This outcome highlights the increased sensitivity of higher quantile values to outliers, which typically correspond to deeper ponds. It also demonstrates the need for the upper cap of $q^{*}=0.95$ imposed in Eqn (3). Consequently, depth estimates in the deepest ponds, that is, those exceeding 2.35 m in maximum depth, may exhibit greater uncertainty.

## 5. Summary and conclusions

The analysis in this paper addresses the lack of sea-ice melt-pond-depth datasets, particularly those with Arctic- and Antarctic-wide coverage, which represent a critical variable for understanding sea-ice evolution, especially in the context of an imminent transition from perennial to seasonal Arctic sea-ice cover. The ATLAS sensor aboard NASA’s ICESat-2 Mission was originally designed to provide observations of ice-surface heights over land ice and sea ice. Close analysis of the data after launch revealed that for data collected over Arctic sea ice during the melt season, the ATL03 geolocated photon point cloud often includes returns from secondary surfaces, likely corresponding to the bottoms of melt ponds. These secondary returns can not only introduce errors in the surface height freeboard reported by ICESat-2 ATLAS sea-ice data products—such as ATL07, which provides a single surface height (Farrell and others, 2020; Kwok and others, 2022)—but more importantly, they highlight the potential to extract information about melt-pond locations and depths. To leverage this potential, the DDA-bifurcate-seaice algorithm was developed to automatically detect melt pond locations without requiring *a priori* information and to retrieve melt-pond depths using ATL03 data (Herzfeld and others, 2023). In this paper, we utilize Chiroptera lidar and image data from the 2022 NASA ICESat-2 Project Arctic Summer Sea Ice Campaign to assess and evaluate the detection capabilities of the DDA-bifurcate-seaice, and for optimization of the algorithm-specific parameter that controls depth determination, aiming to reduce existing uncertainties in depth estimation.

As a first result, we demonstrate that the DDA-bifurcate-seaice algorithm can automatically detect and accurately characterize a wide range of sea-ice melt ponds, which we validate through comparisons with Chiroptera data. This includes ponds located adjacent to ice ridges, ponds with highly irregular shapes, and—critically—ponds at various stages of melt that exhibit diverse bottom morphologies and mixtures of meltwater and sea water.

A second result of our analysis identifies an optimal melt-pond-depth quantile for retrieving pond-bottom surface heights. We determine this value by precisely aligning Chiroptera-515 lidar data with drift-corrected ICESat-2 observations over sea-ice melt ponds surveyed during the campaign. We run the DDA-bifurcate-seaice algorithm across a range of depth quantile parameter values ($q_{d}$), each producing a distinct estimate of the pond-bottom surface. These estimates are evaluated by minimizing the MSE relative to Chiroptera-515 bottom surface height estimates. Our results indicate that the optimal $q_{d}$ value depends on the maximum pond depth. To address this variability, we derive a piecewise linear function, the depth-quantile function (Eqn (3)), that dynamically adjusts $q_{d}$ based on an initial depth estimate obtained using a fixed $q_{d}$ value, thereby enabling dynamic quantile selection within the DDA-bifurcate-seaice framework.

Results indicate that when prescribing a global and uniform depth quantile, a value of qd=0.75 is optimal, supporting its use in release 7 of the ATL07 data product. Comparing to Chiroptera estimates, the use of qd=0.75 globally results in pond depth estimates biased only 0.083 m deeper with a MSE of 0.22 m${}^{2}$.

Implementation of the depth-quantile function reduces bias and MSE in depth estimates relative to Chiroptera measurements by factors of approximately 2.75 and 2.2, respectively, compared to the global, uniform setting of qd=0.75. These improvements are comparable to those offered by use of the pond-specific, discretely optimized melt-pond-depth parameter. The enhancement given by the depth-quantile function improves the overall accuracy and reliability of depth estimates from the DDA-bifurcate-seaice algorithm, leading to reduced uncertainty. While DDA-bifurcate-seaice depth estimates remain slightly deeper on average, the introduction of the depth-quantile function results in a reduction of the mean depth bias from 0.083 m to 0.030 m and the mean MSE from 0.22 to 0.10 m${}^{2}$ across all ponds analyzed.

Our analysis optimizes melt-pond-depth estimates to the extent permitted by the evaluation dataset, which is based on Chiroptera-515 airborne lidar measurements and is largely confined to a region of multi-year sea ice. Notably, melt-pond-depth retrieval over multi-year ice represents the more challenging case relative to first-year ice, as multi-year ice exhibits a wide range of pond-bottom morphologies, from smooth to highly rough surfaces, as illustrated by the example ponds, thereby providing a more stringent testbed for optimization and coincident measurement analyses. Further reductions in ICESat-2/DDA depth uncertainty are constrained by the inherent uncertainties of the Chiroptera data itself, as well as the small sample size of 10 melt ponds used in this study. A more comprehensive assessment of uncertainty will therefore require a larger and more diverse dataset, which may be enabled by incorporating data from future ICESat-2-based airborne validation campaigns that include both multi-year and first-year ice regions, or by developing a broad curated dataset of coincident satellite measurements.

Melt pond information derived from NASA ICESat-2 data using the DDA-bifurcate-seaice algorithm is now included in release 7 of the ATLAS/ICESat-2 Sea-Ice Melt-Pond Product, as an experimental component (Kwok and others, 2025). Results from the depth optimization derived in this paper may be used to inform the release 8 data product. More generally, the work presented here is expected to lay the foundation not only for improved algorithms supporting a future ICESat-2 melt-pond product, but also for high-resolution detection and height/depth retrieval of surface and subsurface features in data from future lidar altimeters and similar remote sensing instruments.

## Supporting information

10.1017/jog.2026.10167.sm001 Trantow et al. supplementary material Trantow et al. supplementary material

## Data Availability

The ICESat-2 ATL03 (Global Geolocated Photon Data) and ATL07 (Sea Ice Height) datasets used in this study are publicly available from the National Snow and Ice Data Center (NSIDC). The datasets can be accessed and downloaded via the NSIDC data access tool (nsidc.org/data/data-access-tool) or through NASA’s Earthdata platform (https://earthdata.nasa.gov). Users must register for an Earthdata account to access and download the data. The Chiroptera lidar dataset is available to the public at Texas Data Repository: https://doi.org/10.18738/T8/MYOUUV.

## Supplementary material

The supplementary material to this paper can be found on https://doi.org/10.1017/jog.2026.10167.

## Figure 1 Long description

The image A shows a map of Greenland with a flight path marked as a solid red line labeled 'ICESat-2 RGT 531' and 'Flight path 2022-07-26'. The map highlights Thule Air Base and Pituffik Space Base. A gray box indicates the location of sub-image B. The image B shows a zoomed-in view of the flight path with color-coded data segments labeled FL1 to FL1.5B. The dashed magenta line represents the ICESat-2 RGT 531 path, while the solid lines in various colors represent different data segments from the flight on 26 July 2022.

Navigate back to Figure 1.

## Figure 2 Long description

A flowchart titled 'DDA-bifurcate-seaice Algorithm Steps' shows six main processes. The input is 'ICESat-2 ATL03 Geolocated Photon Data'. The steps are: (1) Large-scale separation of noise and signal slabs, labeled 'l'. (2) Photon density calculation, labeled 's, u, a'. (3) Auto-adaptive thresholding, labeled 'k, q'. (4) Peak identification in along-track elevation histograms, labeled 'z, x, M, O'. (5) Melt-pond-specific ground follower, labeled 'R, r, Q, S, qd'. (6) Correction of melt-pond shapes and removing false positives, labeled 'md, mw'. The output is 'Height of surface(s), pond width, mean pond depth, maximum pond depth, saturation characteristics'. Arrows indicate the flow from one step to the next.

Navigate back to Figure 2.

## Figure 3 Long description

The image contains six graphs illustrating photon data analysis. The first graph shows raw photon data for Segment 5, with a scatter plot of photon events. The second graph displays noise and signal separation, with noise in red and signal in green. The third graph presents the density dimension of thresholds along the track, with various photon categories marked. The fourth graph shows signal photons colored by resolution, with a color scale indicating density. The fifth graph illustrates density dimension along the track for Pass 0, with a color gradient representing density. The sixth graph provides the final ground estimate for both passes, with a color scale indicating density and signal photons marked.

Navigate back to Figure 3.

## Figure 4 Long description

The image A shows a graph titled 'Pond 3775 (Flight Segment: 2022-07-26 FL1 SC RGT: 531 Beam: gt2r)' with x-axis labeled 'y-position (m)' and y-axis labeled 'Depth (m)'. It includes various data points and lines representing different DDA top surfaces and Chiroptera data. The image B shows an aerial view of Pond-3775 with a green line indicating the ICESat-2 survey path across the pond width. The image C shows a graph titled 'Pond 3675 (Flight Segment: 2022-07-26 FL1 SC RGT: 531 Beam: gt2r)' with similar axes and data representation as the first graph. The image D shows an aerial view of Pond-3675 with a similar green line indicating the ICESat-2 survey path. Both graphs and images illustrate the pond depths and survey paths using Chiroptera and ICESat-2 data.

Navigate back to Figure 4.

## Figure 5 Long description

The image A shows Pond-3775 with survey paths marked by lines. The red line indicates the survey path, the blue line shows the DDA-determined pond width and the green line represents the ICESat-2 footprint. The image B displays a graph titled 'Pond 3775 (Flight Segment: 2022-03-28 11:52, RGT: 531, Beam: gt3l)' with x-axis labeled 'y position (m)' and y-axis labeled 'height (m)'. It shows ICESat-2/DDA photon classification with red, blue and green points. The image C presents a graph titled 'Pond 3775 (Flight Segment: 2022-03-28 11:52, RGT: 531, Beam: gt3l)' with x-axis labeled 'y position (m)' and y-axis labeled 'height (m)'. It shows Chiroptera-515 photons within a 2 m radius of the ICESat-2 survey line, marked in green. The image D shows a graph titled 'Pond 3775 (Flight Segment: 2022-03-28 11:52, RGT: 531, Beam: gt3l)' with x-axis labeled 'y position (m)' and y-axis labeled 'height (m)'. It displays ICESat-2 photons weighted by density, with points colored from orange to blue. The image E presents a graph titled 'Pond 3775 (Flight Segment: 2022-03-28 11:52, RGT: 531, Beam: gt3l)' with x-axis labeled 'y position (m)' and y-axis labeled 'height (m)'. It shows pond depths with various depth quantiles, with the Chiroptera-515 bottom surface estimate marked by a green line. The image F displays a graph titled 'Pond 3775 (Flight Segment: 2022-03-28 11:52, RGT: 531, Beam: gt3l)' with x-axis labeled 'y position (m)' and y-axis labeled 'height (m)'. It shows optimal depth given by a purple line, comparing DDA-bifurcate-seaice and Chiroptera-515 photon distributions and surface heights for various depths over Pond-3775.

Navigate back to Figure 5.

## Figure 6 Long description

The image contains six sub-images labeled a to f. Sub-image a shows Pond-2535 with a survey path marked by a red line, pond width by a blue line and an 11 meter footprint by a green line. Sub-image b presents a graph of Pond-2535 with the x-axis labeled 'y position (m)' and the y-axis labeled 'height (m)'. It includes various photon distributions and surface heights. Sub-image c shows another graph for Pond-2535 with similar axes, focusing on optimal depth. Sub-image d displays Pond-3248 with similar survey markings as sub-image a. Sub-image e provides a graph for Pond-3248 with the same axes as sub-image b, showing photon distributions. Sub-image f presents a graph for Pond-3248 focusing on optimal depth, similar to sub-image c. Each graph includes plotted values and lines representing different depth estimates and photon distributions.

Navigate back to Figure 6.

## Figure 7 Long description

The image contains six sub-images labeled a to f. Sub-image a shows Pond-609 with a survey path across the pond marked by a red line, the DDA-determined pond width by a blue line and the extent of the 11 meter footprint of ICESat-2 in green. Sub-image b displays ICESat-2/DDA photon classification after thresholding, with altitude on the y-axis and SP latitude on the x-axis. Sub-image c shows Chiroptera-515 photons within a 2 meter radius of the ICESat-2 survey line, with altitude on the y-axis and SP latitude on the x-axis. Sub-image d presents ICESat-2 photons weighted by density, with altitude on the y-axis and SP latitude on the x-axis. Sub-image e illustrates pond depths with various depth quantiles, with the Chiroptera-515 bottom surface estimate marked by a green line and altitude on the y-axis and SP latitude on the x-axis. Sub-image f shows optimal depth given by a purple line, with altitude on the y-axis and SP latitude on the x-axis. Each graph includes labeled axes and plotted data points, with legends indicating different data sets and measurements.

Navigate back to Figure 7.

## Figure 8 Long description

The image contains two main sections, each with a pond image and two graphs. The first section (a-c) shows Pond-705. Image (a) displays the pond with a survey path marked by a red line, pond width by a blue line and an 11-meter footprint of ICESat-2 in green. Graph (b) presents depth data with various quantiles and a Chiroptera-515 bottom surface estimate in green. Graph (c) shows optimal depths with a purple line. The second section (d-f) shows Pond-738. Image (d) displays the pond with similar markings as Pond-705. Graph (e) presents depth data with various quantiles and a Chiroptera-515 bottom surface estimate in green. Graph (f) shows optimal depths with a purple line. Both sections include photon distributions and surface heights for various depths.

Navigate back to Figure 8.

## Figure 9 Long description

The image contains two sets of images and graphs labeled a to f. Image a shows Pond-3675 with a survey path marked by a red line, pond width by a blue line and an 11-meter footprint of ICESat-2 in green. Image d shows Pond-3273 with similar markings. Graph b displays depth data for Pond-3675 with various depth quantiles and a Chiroptera-515 bottom surface estimate. Graph e shows similar data for Pond-3273. Graph c presents optimal depths for Pond-3675, while graph f shows optimal depths for Pond-3273. Each graph includes photon distributions and surface heights for different depths.

Navigate back to Figure 9.

## Figure 10 Long description

The image contains two graphs. The first graph (a) plots maximum melt-pond depth in meters on the y-axis against optimal depth quantile on the x-axis. It includes data points for maximum depths at qd equals qd subscript opt and qd equals 0.75, with linear fits for each. The second graph (b) shows a depth-quantile function with qd superscript star on the y-axis and d subscript 75, max in meters on the x-axis, depicting a linear relationship.

Navigate back to Figure 10.

## Table 1 Long description

The table details parameters used in the ICESat-2 Summer 2022 Arctic Sea Ice Campaign, focusing on the DDA-bifurcate-seaice algorithm. Key parameters include a standard deviation of 3 meters for the kernel, a cutoff at two standard deviations, and an anisotropy value of 5. Notably, the horizontal histogram bin size ranges from 25 to 70 meters, updated for improved melt-pond detection. The resolution of the ground follower is set at 5 meters, and the minimum peak height and prominence in histograms are both set at 2. The melt-pond depth quantile is 0.75, with a minimum depth of 0.5 meters required for identification. These parameters are crucial for accurately capturing sea-ice melt pond characteristics.

Navigate back to Table 1.

## Table 2 Long description

The table presents optimization results for depth quantile parameters of ten sea-ice melt ponds from the FL1 flight segment on July 26, 2022. Each pond is identified by a unique ID and associated with specific figures. The surface height varies, with pond 4311 having the lowest at 0.418 meters, and pond 3675 the highest at 0.510 meters. Maximum depths at a fixed quantile of 0.75 and at optimal quantiles are compared, showing pond 609 with the greatest depth at optimal quantile, 2.223 meters. Ponds are ordered by their ICESat-2 measurement time, with pond 4311 recorded earliest and pond 609 latest. The optimal depth quantile varies significantly, indicating diverse pond characteristics and measurement conditions.

Navigate back to Table 2.

## Table 3 Long description

The table compares depth determination differences using a fixed quantile parameter and an optimized one for melt ponds. It reports bias and mean squared error for each method, along with differences in maximum and mean depth estimates. Pond 705 exhibits the largest pointwise depth difference, while Pond 4311 shows the smallest bias with optimized quantile values. Overall, optimized quantile parameters generally reduce bias and mean squared error, indicating improved depth estimation accuracy. The average optimized quantile parameter is 0.763, with varying impacts on depth estimates across different ponds. Interpretation should consider the variability in pond-specific quantile values and their effect on depth estimation accuracy.

Navigate back to Table 3.

## Table 4 Long description

The table compares depth determination differences using fixed and adaptive quantile methods for melt-pond-depth estimation. It lists the improved quantile, optimal quantile, bias, mean squared error, and depth differences for each pond. Adaptive quantiles generally result in lower bias and mean squared error compared to fixed quantiles. Notable findings include Pond 3775, where adaptive quantiles drastically reduce bias and error, and Pond 705, which shows the largest absolute pointwise depth difference. The average values indicate that adaptive quantiles improve overall accuracy, but individual pond results vary, highlighting the importance of context-specific quantile selection.

Navigate back to Table 4.
