# Supplementary material for: Optimization of automated sea-ice melt-pond-depth determination in ICESat-2 altimeter data with the Density-Dimension Algorithm for bifurcating sea-ice reflectors using airborne campaign data
Source: J Glaciol. 2026 May 19;72:e53. doi: 10.1017/jog.2026.10167 (PMC13320595; doi:10.1017/jog.2026.10167)
Supplement: Trantow et al. supplementary material [file S0022143026101671sup001.pdf]

Supplementary Material for Manuscript Titled: "Optimization of automated sea-ice melt-pond depth determination in ICESat-2 altimeter data with the Density-Dimension Algorithm for bifurcating sea-ice reflectors using airborne campaign data"

Thomas Trantow [1,*], Ute C. Herzfeld [1,2], Mia Vanderwilt[3,4], Kutalmis Saylam[5], Nathan Kurtz[4], Huilin Han[1] and Rachel Tilling[3]

[1] Geomathematics, Remote Sensing and Cryospheric Sciences Laboratory; Department of Electrical, Energy and Computer Engineering; University of Colorado, Boulder, Colorado, USA

[2] Department of Computer Science; University of Colorado, Boulder, Colorado, USA

[3] Earth System Science Interdisciplinary Center, University of Maryland, College Park, MD, USA

[4] NASA Goddard Space Flight Center, Greenbelt, Maryland, USA

[5] Bureau of Economic Geology, Near Surface Observatory, Jackson School of Geosciences, University of Texas, Austin, Texas, USA

[*] Corresponding author

S1 Full results of melt pond depth optimization procedure

Table S1 provides the Mean Squared Error (MSE) measure between Chiroptera-515 and ICESat-2/DDA melt pond depths for each melt pond and each qd-parameter value, following the procedure described in Section 3.3 of the main manuscript. The minimized value across all qd values, i.e. qd_{opt}, is indicated by the boldface value for each pond in the table.

**Pond ID**	**0.0**	**0.1**	**0.2**	**0.3**	**0.4**	**0.5**	**0.6**	**0.7**	**0.75**	**0.8**	**0.9**	**0.95**	**0.98**	**1.0**
4311	0.89	0.26	0.069	0.049	**0.043**	0.10	0.10	0.11	0.12	0.14	0.17	0.21	0.23	0.25
4108	4.72	3.64	1.44	0.30	0.090	**0.042**	0.062	0.12	0.12	0.12	0.11	0.11	0.11	0.11
3775	3.32	2.15	1.52	1.12	0.80	0.63	0.44	0.35	0.27	0.21	0.10	**0.080**	0.097	0.13
3675	0.69	0.39	0.29	0.18	0.18	0.17	0.15	0.15	0.16	0.16	0.092	**0.086**	0.088	0.090
3273	1.76	0.84	0.55	0.33	0.24	0.11	**0.062**	0.067	0.082	0.10	0.12	0.13	0.15	0.15
3248	0.93	0.55	0.37	0.30	0.19	**0.163**	0.20	0.22	0.21	0.20	0.17	0.17	0.168	0.165
2535	1.74	1.74	1.34	1.08	0.87	0.74	0.46	0.30	0.23	0.19	0.13	0.107	**0.1009**	0.1017
738	0.63	0.50	0.41	0.29	0.19	0.12	0.073	0.043	0.038	**0.037**	0.046	0.049	0.052	0.054
705	3.75	2.77	2.13	1.70	1.22	0.84	0.70	0.50	0.41	0.34	0.22	0.187	0.176	**0.174**
609	1.37	1.06	0.83	0.60	0.48	0.40	0.31	0.26	0.22	0.20	0.17619	**0.17612**	0.18	0.19

Table S1. Mean Squared Error between depths provided by the DDA-bifurcate-seaice with varying melt-pond quantile values (qd). Normalized by number of depth estimates per pond. Numbers in the column headers refer to melt-pond depth quantile parameter (qd) values. Remaining DDA parameters for all runs here are given by Table 1 in the main manuscript. Boldface values denote optimized melt pond quantile value, $qd = qd_{opt}$, for the each pond given by the minimum MSE measure.

S2 Total drift and height correction for each melt-pond in the depth optimization analysis.

Table S2 below provides the exact drift corrections applied to the ICESat-2 data associated with each of the 10 ponds from the analysis in both the x and y directions. In addition, we provide the exact height, or z, correction applied to the ICESat-2 data when matching pond surface heights to Chiroptera-515 data.

**Pond ID**	**Flight Segment**	Δx **(m)**	Δy **(m)**	Δz **(m)**
4311	FL1-SC	-15	-30	-17.22
4108	FL1-SC	-11	-32	-17.24
3775	FL1-SC	-16	-32	-17.15
3675	FL1-SC	-10	-40	-17.11
3273	FL1-SC	-2	-31	-17.074
3248	FL1-SC	-6	-40	-17.07
2535	FL1-SB	-5	-30	-17.165
738	FL1-SB	13	-12	-16.695
705	FL1-SA	8	-16	-16.695
609	FL1-SA	14	-11	-16.68

Table S2. Total drift and surface-height correction for each melt-pond used in the depth analysis. Horizontal corrections in meters applied to polar-stereographic coordinates of the ICESat-2/DDA output data. Vertical corrections, also in meters, and also applied to ICESat-2/DDA output data to match the melt-pond surface height of the Chiroptera-515 data.

S3 Full plot sequences of additional individual ponds

Pond-2535 is another example of a clear melt pond with a smooth bottom surface, but one that is also clearly partially drained (Figure S1). Its connection to the underlying sea-water is not obvious, but may be related to the various dark, circular features distributed throughout the pond. The ICESat-2 transect stretches 75 m and has an optimized depth quantile of $qd_{opt} = 0.98$. Its maximum depth with $qd = 0.75$ is 2.259 m while with $qd_{opt} = 0.98$ that maximum depth is adjusted lower to 2.020 m. Pond-2535 has a top surface height of 0.309 m.

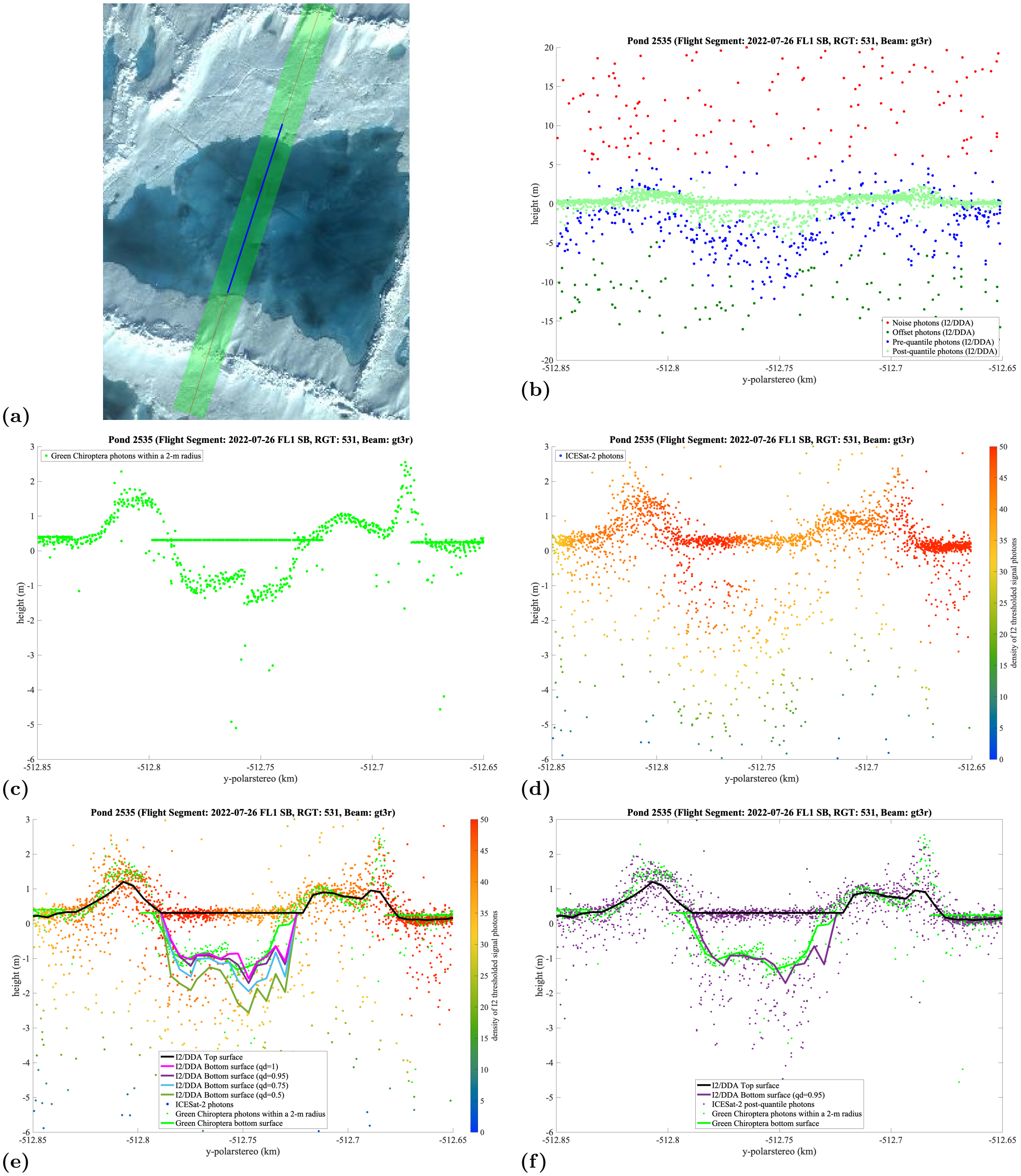

Figure S1. DDA-bifurcate-seaice and Chiroptera-515 photon distributions and surface heights for various depths over Pond-2535. (a) Pond-2535 in Chiroptera imagery with survey path across the pond given by the red line, the DDA-determined pond width by the blue line, and the extent of the 13 m footprint of ICESat-2 in green. (b) ICESat-2/DDA photon classification based after the thresholding procedure. (c) Chiroptera-515 photons within a 2 m radius of ICESat-2 survey line. (d) ICESat-2 photons weighted by density. (e) Pond depths with various depth quantiles (qd) with the Chiroptera-515 bottom surface estimate (green line). (f) Optimal depth given by $qd = 0.95$ (purple line).

Pond-3248 provides another example of a clear melt pond with a smooth bottom while also being relatively shallow (Figure S2). Its oblong shape and proximity to a long sea-ice ridge along its western flank make its characterization difficult but not impossible if one uses the DDA-bifurcate-seaice with proper parameterization. The ICESat-2 transect across the length of the pond is 60 m. Maximum depth estimates are 0.912 m with $qd = 0.75$ and 1.124 m using its optimized value of $qd_{opt} = 0.5$. The height of its top surface is 0.502 m.

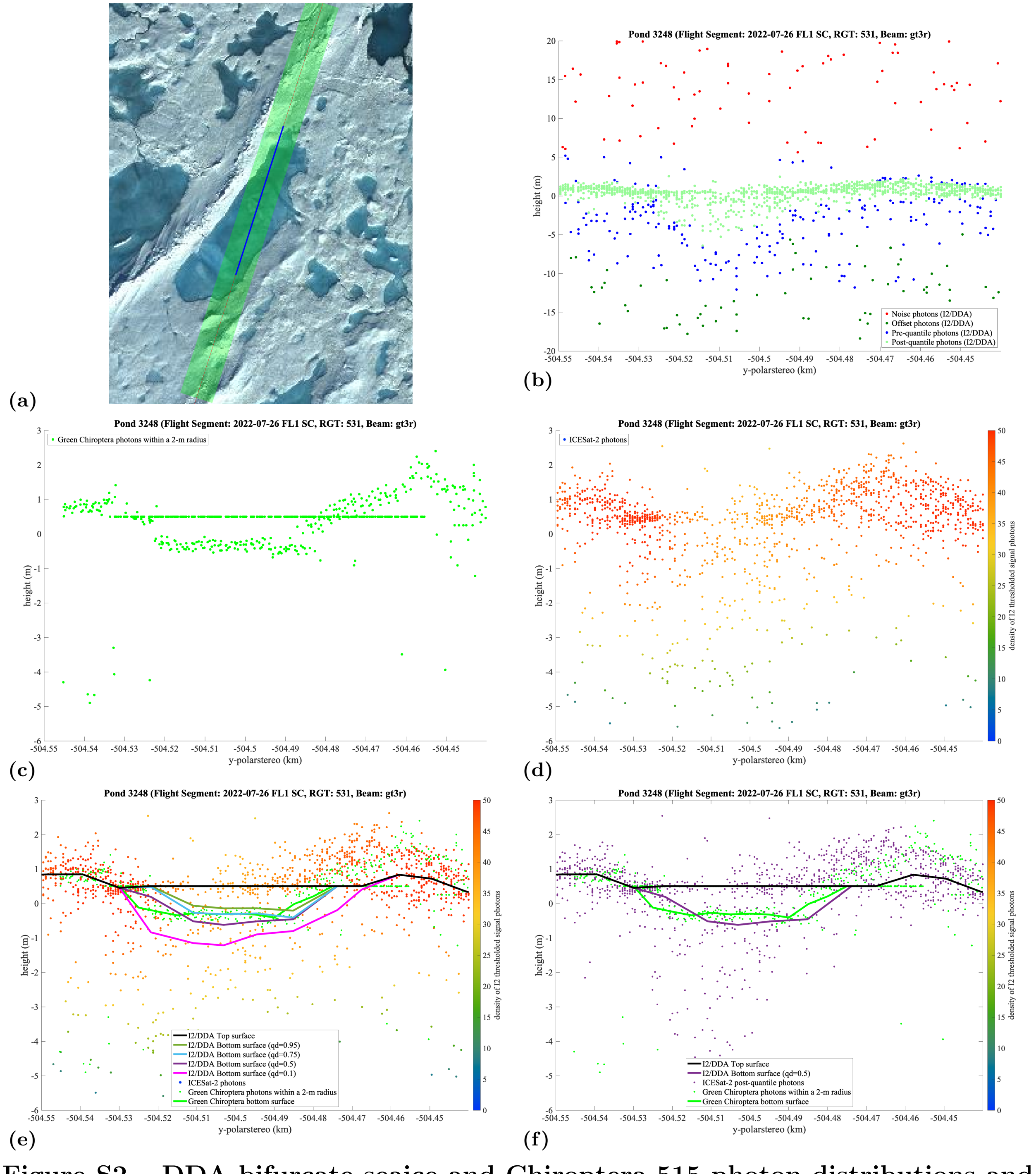

Figure S2. DDA-bifurcate-seaice and Chiroptera-515 photon distributions and surface heights for various depths over Pond-3248. (a) Pond-3248 in Chiroptera imagery with survey path across the pond given by the red line, the DDA-determined pond width by the blue line, and the extent of the 13 m footprint of ICESat-2 in green. (b) ICESat-2/DDA photon classification based after the thresholding procedure. (c) Chiroptera-515 photons within a 2 m radius of ICESat-2 survey line. (d) ICESat-2 photons weighted by density. (e) Pond depths with various depth quantiles (qd) with the Chiroptera-515 bottom surface estimate (green line). (f) Optimal depth given by $qd = 0.5$ (purple line).

Figures S3–S4 (Ponds 705 and 738) illustrate additional examples of dark ponds with rough bottoms, while Figures S5–S6 (Ponds 3675 and 3273) show partially drained ponds, and are presented here in their complete plot sequences. Detailed descriptions and analyses of these ponds are provided in the main manuscript, along with their corresponding abbreviated plots (Figs. 8 and 9).

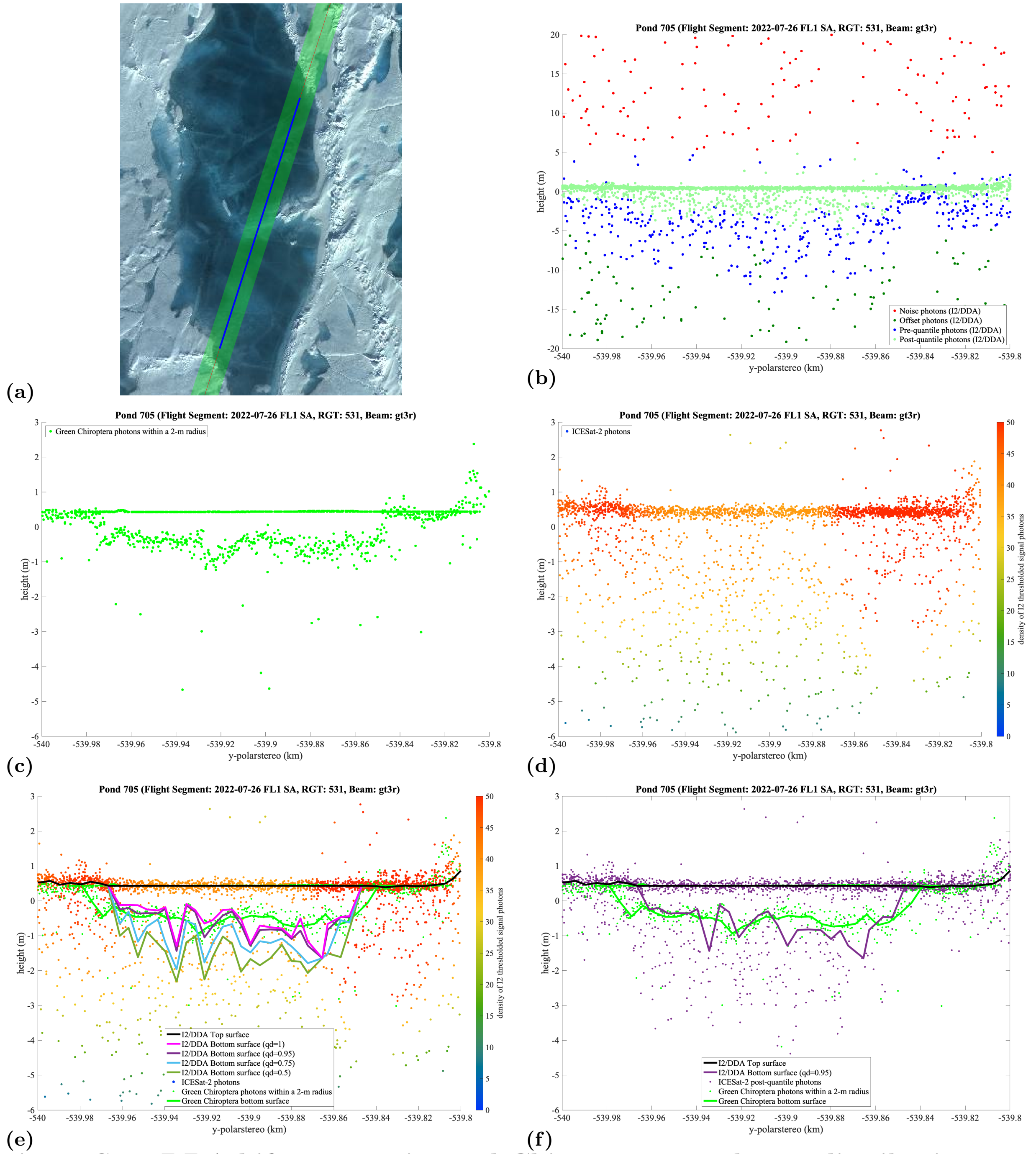

Figure S3. DDA-bifurcate-seaice and Chiroptera-515 photon distributions and surface heights for various depths over Pond-705. (a) Pond-705 in Chiroptera imagery with survey path across the pond given by the red line, the DDA-determined pond width by the blue line, and the extent of the 11 m footprint of ICESat-2 in green. (b) ICESat-2/DDA photon classification based after the thresholding procedure. (c) Chiroptera-515 photons within a 2 m radius of ICESat-2 survey line. (d) ICESat-2 photons weighted by density. (e) Pond depths with various depth quantiles (qd) with the Chiroptera-515 bottom surface estimate (green line). (f) Optimal depth given by $qd = 0.95$ (purple line).

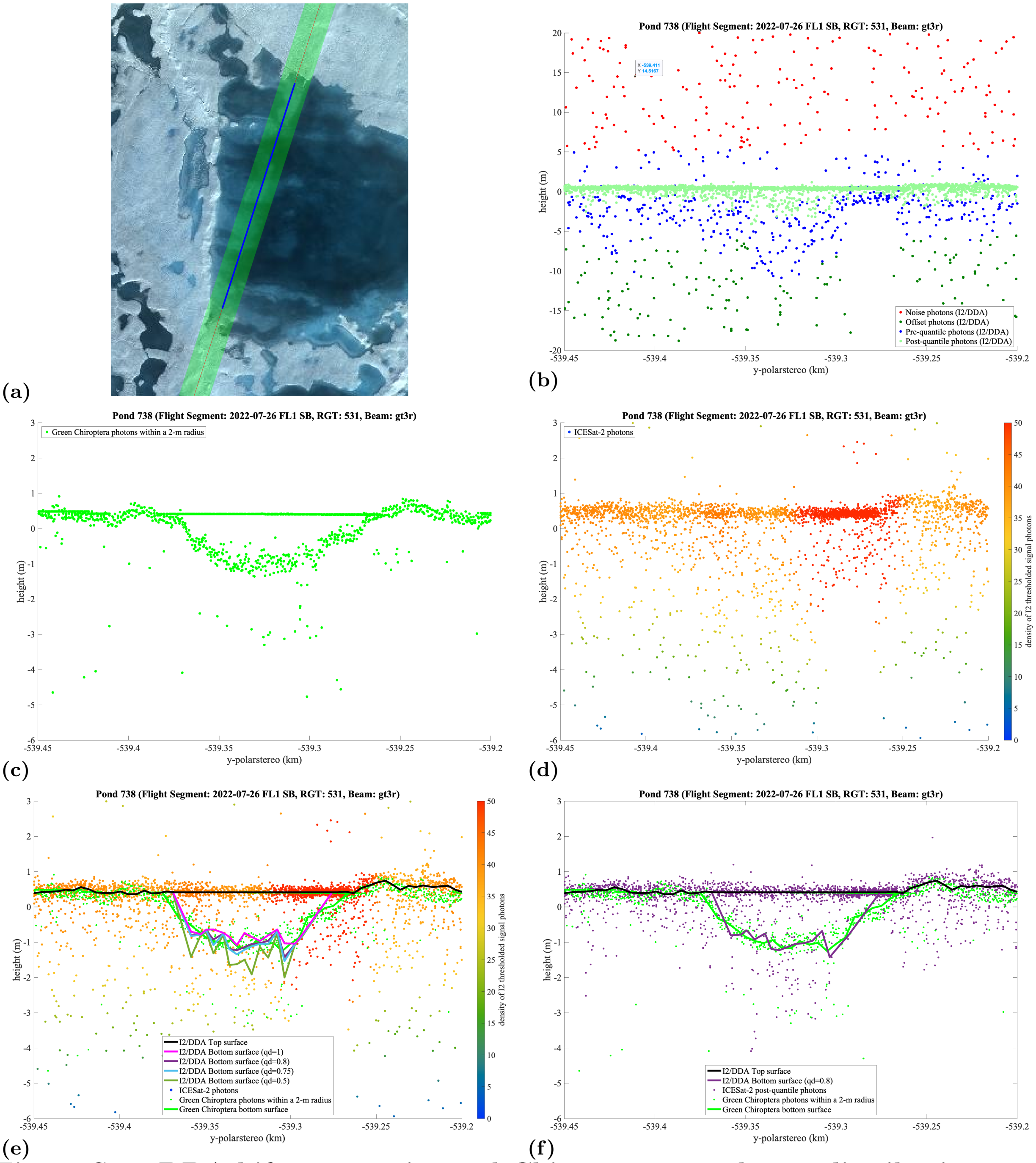

Figure S4. DDA-bifurcate-seaice and Chiroptera-515 photon distributions and surface heights for various depths over Pond-738. (a) Pond-738 in Chiroptera imagery with survey path across the pond given by the red line, the DDA-determined pond width by the blue line, and the extent of the 11 m footprint of ICESat-2 in green. (b) ICESat-2/DDA photon classification based after the thresholding procedure. (c) Chiroptera-515 photons within a 2 m radius of ICESat-2 survey line. (d) ICESat-2 photons weighted by density. (e) Pond depths with various depth quantiles (qd) with the Chiroptera-515 bottom surface estimate (green line). (f) Optimal depth given by $qd = 0.8$ (purple line).

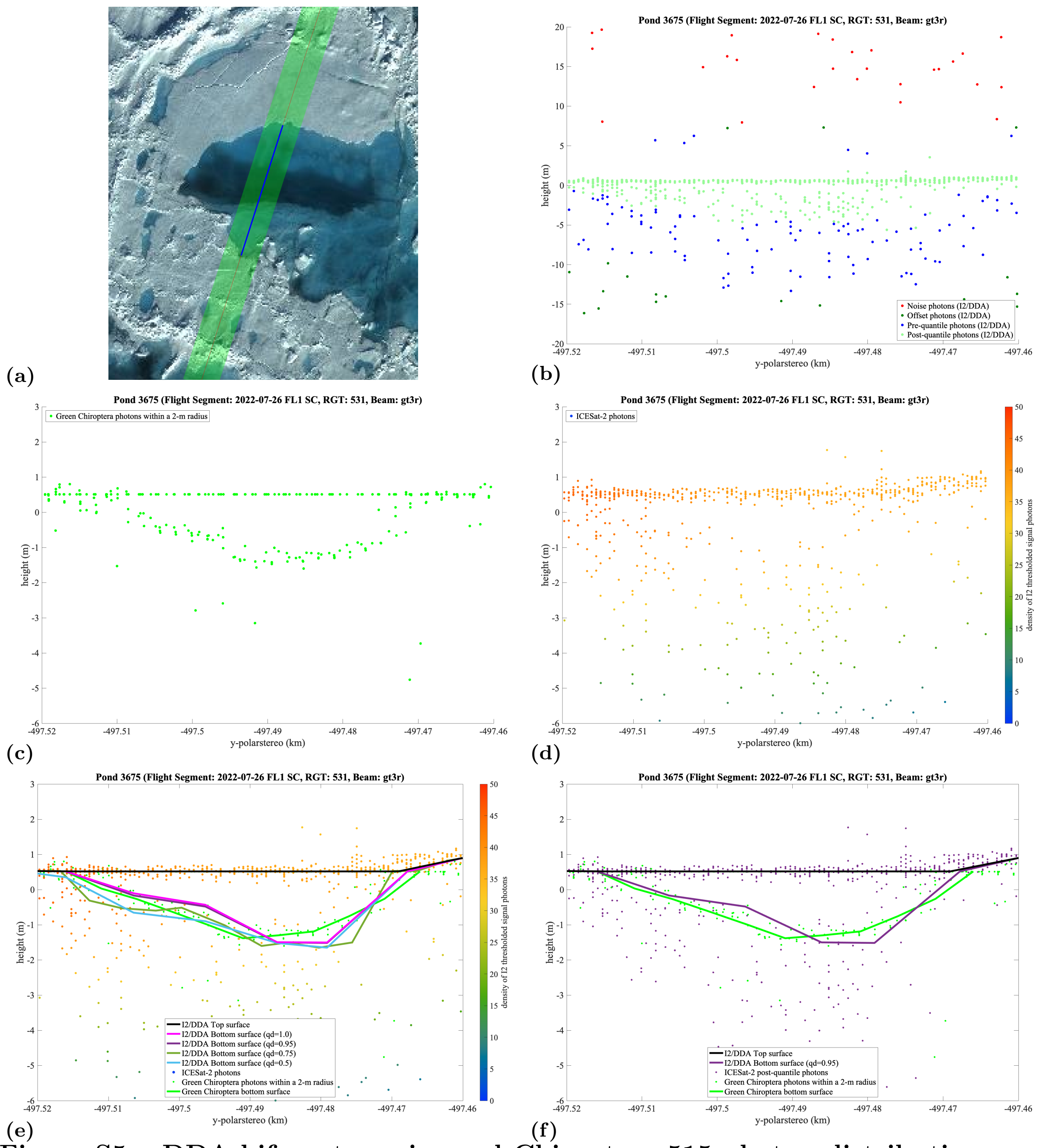

Figure S5. DDA-bifurcate-seaice and Chiroptera-515 photon distributions and surface heights for various depths over Pond-3675. (a) Pond-3675 in Chiroptera imagery with survey path across the pond given by the red line, the DDA-determined pond width by the blue line, and the extent of the 11 m footprint of ICESat-2 in green. (b) ICESat-2/DDA photon classification based after the thresholding procedure. (c) Chiroptera-515 photons within a 2 m radius of ICESat-2 survey line. (d) ICESat-2 photons weighted by density. (e) Pond depths with various depth quantiles (qd) with the Chiroptera-515 bottom surface estimate (green line). (f) Optimal depth given by $qd = 0.95$ (purple line).

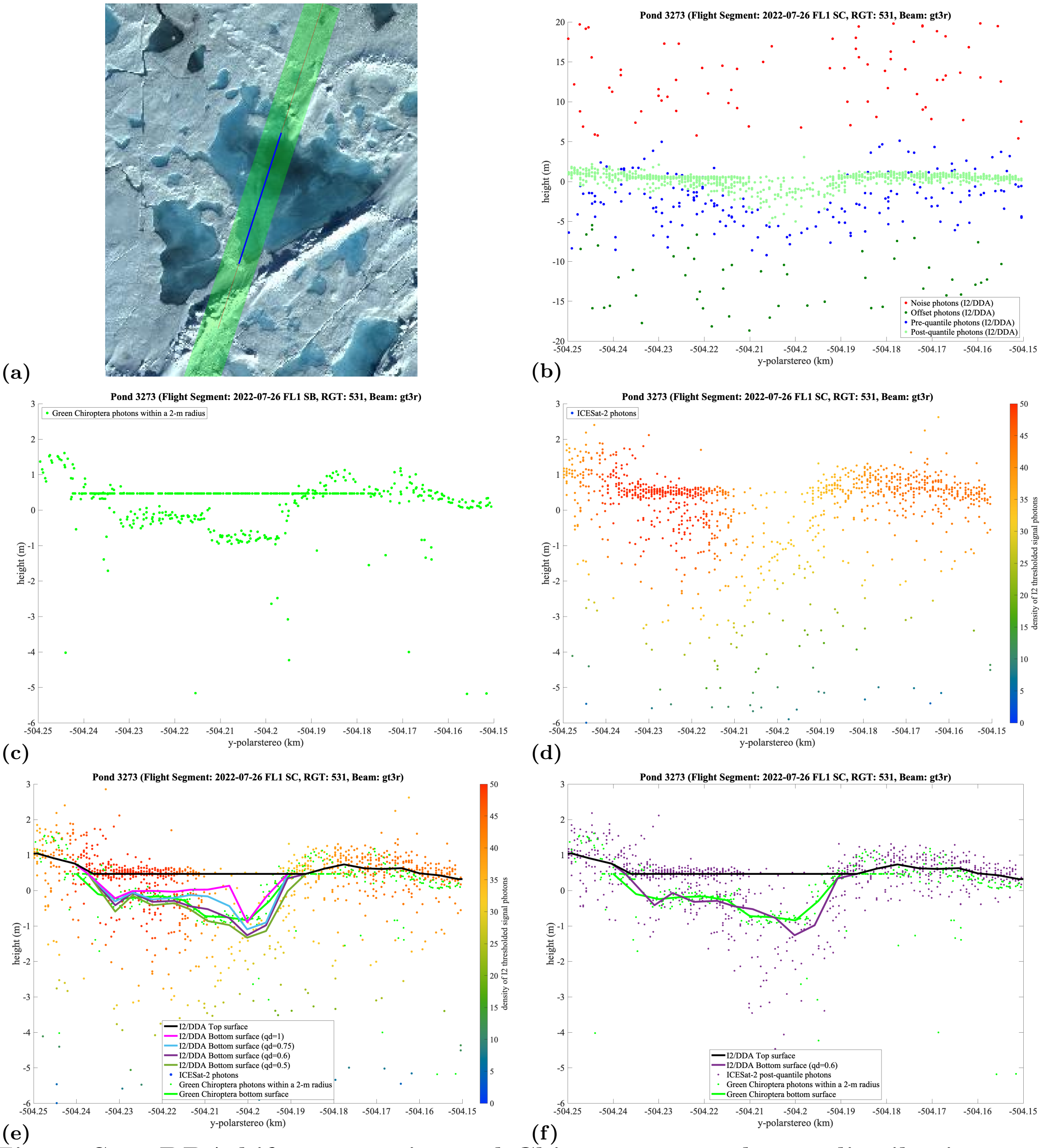

Figure S6. DDA-bifurcate-seaice and Chiroptera-515 photon distributions and surface heights for various depths over Pond-3273. (a) Pond-3273 in Chiroptera imagery with survey path across the pond given by the red line, the DDA-determined pond width by the blue line, and the extent of the 11 m footprint of ICESat-2 in green. (b) ICESat-2/DDA photon classification based after the thresholding procedure. (c) Chiroptera-515 photons within a 2 m radius of ICESat-2 survey line. (d) ICESat-2 photons weighted by density. (e) Pond depths with various depth quantiles (qd) with the Chiroptera-515 bottom surface estimate (green line). (f) Optimal depth given by $qd = 0.6$ (purple line).

Pond-4311 is an example of a shallow pond and a pond with a somewhat rough bottom surface (Figure S7). Features visible in the imagery are reflected in the bottom topography estimate provided by ICESat-2/DDA. The transect length is 35 m with maximum depth estimates of 0.906 m with $qd = 0.75$ and 1.318 m with its optimal value of $qd = 0.4$. Pond-4311 has the lowest qd_{opt} value of the 10 ponds analyzed and is also has the shortest transect length. Finally, this pond has a top surface height of 0.418 m.

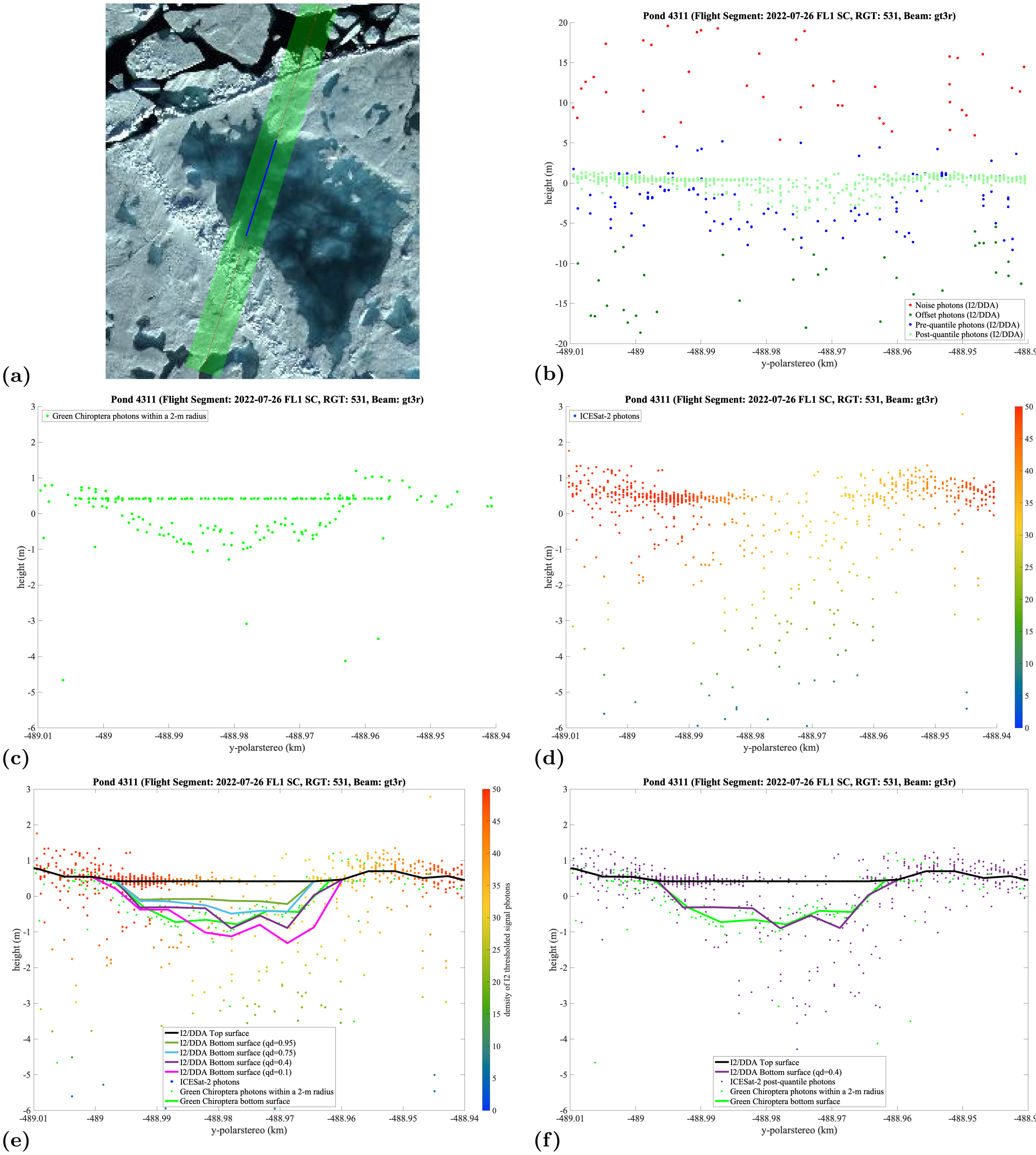

Figure S7. DDA-bifurcate-seaice and Chiroptera-515 photon distributions and surface heights for various depths over Pond-4311. (a) Pond-4311 in Chiroptera imagery with survey path across the pond given by the red line, the DDA-determined pond width by the blue line, and the extent of the 13 m footprint of ICESat-2 in green. (b) ICESat-2/DDA photon classification based after the thresholding procedure. (c) Chiroptera-515 photons within a 2 m radius of ICESat-2 survey line. (d) ICESat-2 photons weighted by density. (e) Pond depths with various depth quantiles (qd) with the Chiroptera-515 bottom surface estimate (green line). (f) Optimal depth given by $qd = 0.4$ (purple line).

Finally, Pond-4108 is another small and shallow pond that shares characteristics with Pond-4311, though its features are less distinct (Figure S8). This pond is relatively dark across its full extent, which implies an extensive linkage with the underlying sea-water. Its bottom topography is not necessarily smooth, consisting of several bumps in the ICESat-2/DDA estimated surface, and therefore the dominant mechanism of melt-water drainage and sea-water connection is not immediately clear. The transect across the center and along the length Pond-4108 is approximately 40 m. The maximum depth estimate when using $qd = 0.75$ is 0.977 m, with this estimate being adjust upward to 1.265 m when using its optimal melt pond quantile of $qd_{opt} = 0.5$. The height of its top surface is 0.351.

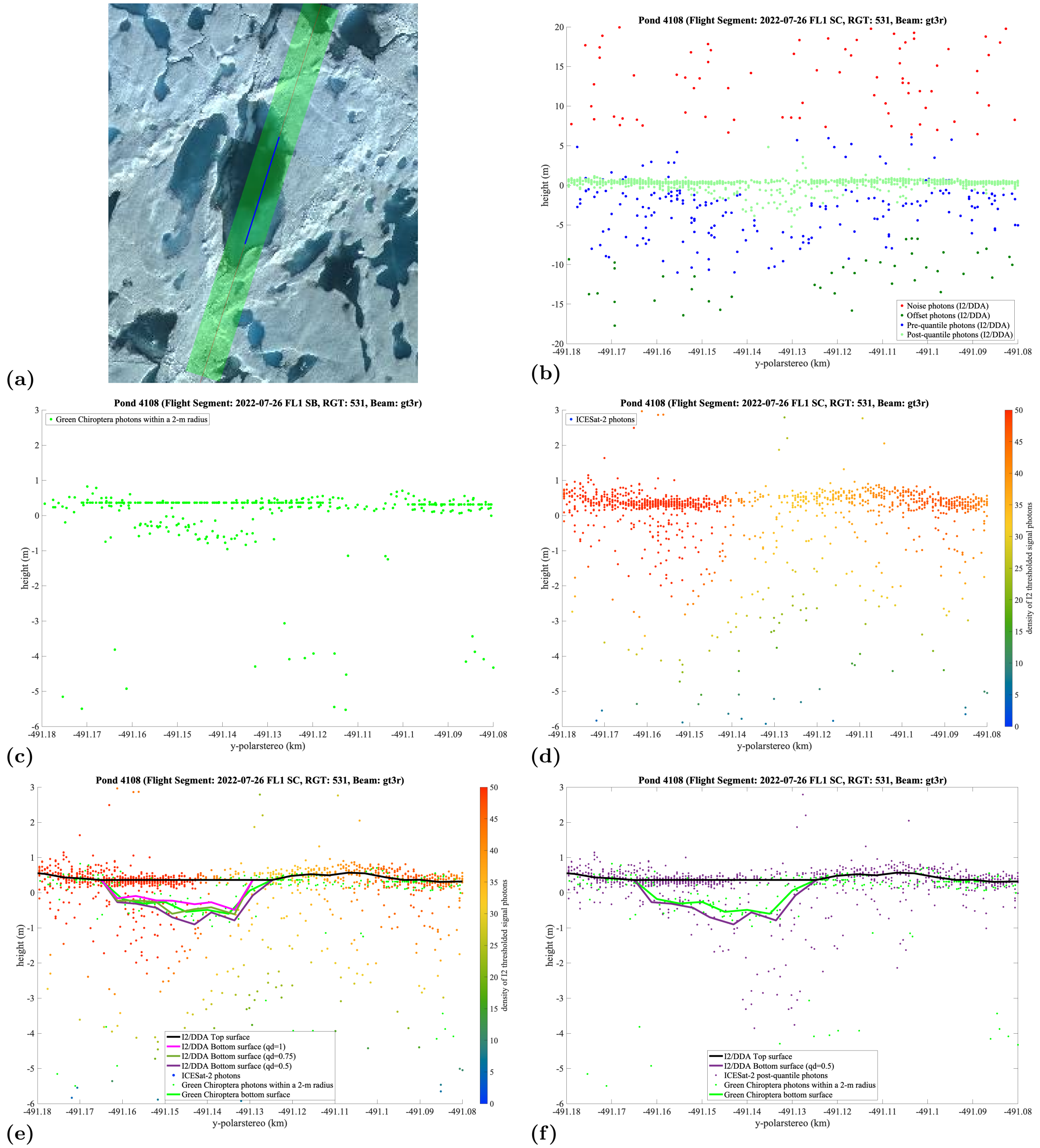

Figure S8. DDA-bifurcate-seaice and Chiroptera-515 photon distributions and surface heights for various depths over Pond-4108. (a) Pond-4108 in Chiroptera imagery with survey path across the pond given by the red line, the DDA-determined pond width by the blue line, and the extent of the 13 m footprint of ICESat-2 in green. (b) ICESat-2/DDA photon classification based after the thresholding procedure. (c) Chiroptera-515 photons within a 2 m radius of ICESat-2 survey line. (d) ICESat-2 photons weighted by density. (e) Pond depths with various depth quantiles (qd) with the Chiroptera-515 bottom surface estimate (green line). (f) Optimal depth given by $qd = 0.5$ (purple line).
